# Structural basis of AMPK regulation by small molecule activators

**DOI:** 10.1038/ncomms4017

**Published:** 2013-12-19

**Authors:** Bing Xiao, Matthew J. Sanders, David Carmena, Nicola J. Bright, Lesley F. Haire, Elizabeth Underwood, Bhakti R. Patel, Richard B. Heath, Philip A. Walker, Stefan Hallen, Fabrizio Giordanetto, Stephen R. Martin, David Carling, Steven J. Gamblin

**Affiliations:** 1 MRC National Institute for Medical Research, The Ridgeway, Mill Hill, London NW7 1AA, UK; 2 MRC Clinical Sciences Centre, Cellular Stress Group, Hammersmith Hospital Campus, Imperial College, DuCane Road, London W12 0NN, UK; 3 Bioscience, CVMD Innovative Medicine Unit, AstraZeneca R&D, Pepparedsleden 1, Mölndal S-43183, Sweden; 4 Medicinal Chemistry, CVMD Innovative Medicine Unit, AstraZeneca R&D, Pepparedsleden 1, Mölndal S-43183, Sweden; 5 These authors contributed equally to this work; 6 Present address: Northern Institute for Cancer Research, Newcastle University, Paul O’Gorman Building Framlington Place, Newcastle Upon Tyne, NE2 4HH, UK; 7 Present address: Medicinal Chemistry, Taros Chemicals GmbH & Co. KG, Emil-Figge-Str. 76a, 44227 Dortmund, Germany

## Abstract

AMP-activated protein kinase (AMPK) plays a major role in regulating cellular energy balance by sensing and responding to increases in AMP/ADP concentration relative to ATP. Binding of AMP causes allosteric activation of the enzyme and binding of either AMP or ADP promotes and maintains the phosphorylation of threonine 172 within the activation loop of the kinase. AMPK has attracted widespread interest as a potential therapeutic target for metabolic diseases including type 2 diabetes and, more recently, cancer. A number of direct AMPK activators have been reported as having beneficial effects in treating metabolic diseases, but there has been no structural basis for activator binding to AMPK. Here we present the crystal structure of human AMPK in complex with a small molecule activator that binds at a site between the kinase domain and the carbohydrate-binding module, stabilising the interaction between these two components. The nature of the activator-binding pocket suggests the involvement of an additional, as yet unidentified, metabolite in the physiological regulation of AMPK. Importantly, the structure offers new opportunities for the design of small molecule activators of AMPK for treatment of metabolic disorders.

## Abstract (web summary)

Small molecule activators of the energy sensing kinase AMPK are promising candidates as therapies for metabolic disease. Xiao *et al*. present the crystal structure of AMPK in complex with a small molecule activator, and show that the drug stabilizes interaction between the catalytic and carbohydrate-binding domains.

## Graphical abstract

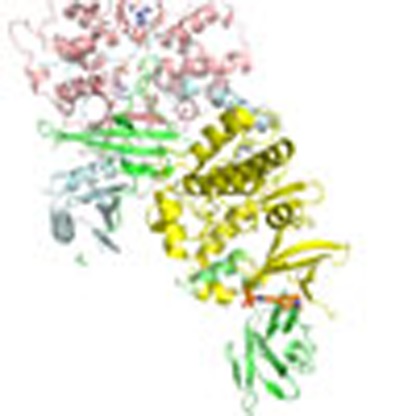

AMP-activated protein kinase (AMPK) plays an important role in regulating energy homeostasis in eukaryotic cells^1^,^2^,^3^. In response to a decrease in cellular ATP levels, for instance following nutrient deprivation or muscle cell contraction, AMPK is activated by phosphorylation of a threonine residue (Thr-172) within the activation loop of the kinase domain^4^. Two upstream kinases, liver kinase B1 (LKB1)^5^,^6^,^7^,^8^ and calcium/calmodulin-dependent protein kinase kinase (CaMKKβ)^9^,^10^,^11^ catalyse phosphorylation of Thr-172 in mammalian cells. Increased AMPK activity leads to a concomitant increase in the phosphorylation of its downstream targets such as acetyl-CoA carboxylase^12^. AMPK phosphorylates a broad range of substrates involved in diverse cellular processes^13^,^14^,^15^,^16^,^17^,^18^,^19^,^20^, but in general terms activation of AMPK leads to a reduction in the rate of anabolic pathways (ATP-utilising) and an increase in the rate of catabolic pathways (ATP-producing)^1^,^2^,^3^. Maintaining a high concentration of ATP relative to ADP is a pre-requisite for eukaryotic cell survival and disturbances in energy homeostasis underlie a wide range of disease states in humans, such as type 2 diabetes and cancer. This key role of AMPK in energy homeostasis makes it an attractive target for the development of drugs aimed at preventing and/or alleviating the detrimental effects of metabolic diseases^15^.

AMPK is a heterotrimeric enzyme complex composed of a catalytic α-subunit, together with β- and γ-regulatory subunits^21^,^22^,^23^,^24^,^25^,^26^. The α-subunit contains an N-terminal protein kinase domain and a C-terminal regulatory domain. The γ-subunit contains four copies of a cystathionine-β-synthase domain^27^, and we previously showed that the γ-subunit binds three molecules of adenine nucleotide^28^. One molecule of AMP is bound tightly and is not exchangeable in solution (Site-4), although soaking studies have suggested that this AMP can be exchanged with ATP in crystalline AMPK^29^,^30^. Nucleotides can bind reversibly to Site-1 and Site-3 in solution, providing a mechanism by which AMPK can respond to changes in ATP levels^28^. Adenine nucleotide binding regulates AMPK activity by three mechanisms. First, AMP causes a two- to threefold allosteric activation^31^,^32^. Second, it has recently been reported that ADP and AMP promote phosphorylation of Thr-172 by LKB1 and CaMKKβ when the β-subunit is myristoylated at its N-terminus^3^,^33^,^34^. Third, ADP and AMP protect Thr-172 against dephosphorylation^35^,^36^,^37^,^38^, and we previously reported the structure of an active form of AMPK phosphorylated on Thr-172, which allowed us to suggest how this mechanism might occur^38^.

In addition to regulation by adenine nucleotides, a number of small molecules have been identified that directly activate AMPK^39^,^40^. The first of these to be reported was A-769662 (refs 39, 41, 42, 43). Two subsequent studies^42^,^43^ showed that activation by A-769662 required the presence of the carbohydrate-binding module (CBM, also known as the glycogen-binding domain) at the N-terminus of the β-subunit, since it shares sequence similarity with a domain found in a number of proteins that bind carbohydrates^44^,^45^. The structure of the isolated CBM from AMPK β1 in complex with β-cyclodextrin has been reported^46^ together with the corresponding region from a yeast analogue as part of a truncated heterotrimer^47^. However, the molecular architecture of drug binding and activation of AMPK has not been determined. The kinase domain of the α-subunit and the CBM of the β-subunit are connected to their C-terminal scaffold domains by flexible linkers. Here we report the crystal structure at 3 Å resolution of AMPK bound to a small molecule activator.

## Results

### Role of carbohydrate-binding module in activator binding

**Figure 1:**
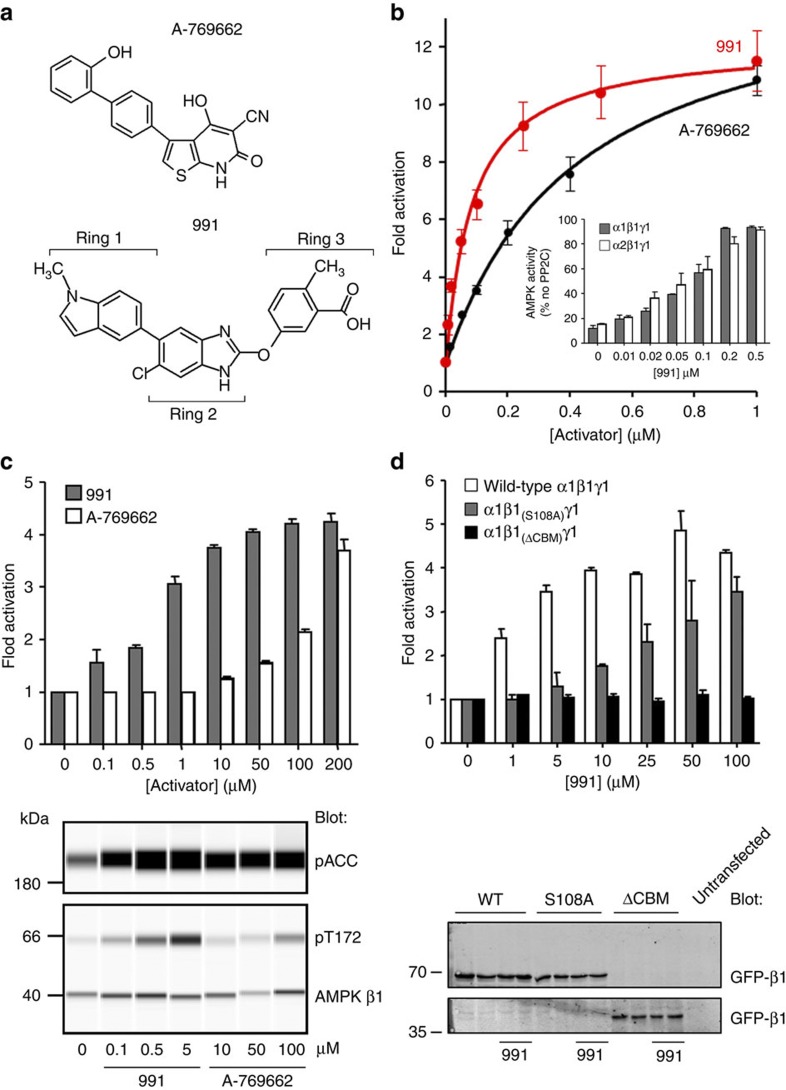
Regulation of AMPK *in vitro* and in HEK293 cells. (**a**) Structures of A-769662 and 991 compounds, the rings of 991 are numbered. (**b**) 991 (red) allosterically activates recombinant α2β1γ1 with half maximal activation (*A*_0.5_) of 0.09±0.02 μM compared to 0.39±0.03 μM for A-769662 (black). Results are the mean±s.e.m. from at least three independent experiments. Inset, 991 protects against pT172 dephosphorylation of α1β1γ1 (grey) and α2β1γ1 (white) complexes. (**c**) Top, 991 (grey bars) activates endogenous AMPK in HEK293 cells at lower doses than A-769662 (white). HEK293 cells were treated with varying concentrations of A-769662 or 991, and endogenous AMPK was immunoprecipitated from cell lysates using a pan-β-specific antibody. AMPK activity was measured using the SAMS peptide assay and results are shown as the fold activation (±s.e.m.) relative to untreated cells from at least three independent experiments. Bottom, 991 and A-769662 increase phosphorylation of endogenous AMPK Thr-172 (pT172) and the AMPK target ACC (pACC) in HEK293 cells (Supplementary Fig. 17). Blots were generated using a capillary-based western blot automated system (Simon, ProteinSimple). (**d**) HEK293 cells were transfected with myc-α1, FLAG-γ1 and either wild-type GFP-β1, GFP-β1 harbouring a mutation at Ser-108 (S108A) or GFP-β1 lacking the CBM (ΔCBM). Cells were treated with varying concentrations of the 991 activator. Top, complexes were immunoprecipitated using the FLAG-tag antibody and AMPK activity measured using the SAMS peptide assay. Results are shown as the fold activity compared with cells not treated with 991 (±s.e.m. from at least three independent experiments). 991 did not activate AMPK lacking the CBM (ΔCBM, black) in HEK293 cells. Higher concentrations of 991 were required to activate the AMPK β1 Ser-108 to alanine mutant (S108A, grey) in HEK293 cells compared with wild-type (white). Bottom, expression of the GFP-β1 subunit was monitored by western blot analysis in cells treated with and without 10 μM 991. An untransfected sample is included as a control.

Our previously published structure of an active AMPK complex contained full-length versions of the α- and γ-subunits but the N-terminal domain of the β-subunit, including the CBM, was engineered out of the construct to facilitate crystallization^38^. Although this construct is allosterically activated by AMP, it is not by A-769662 (ref. 36), and it does not show protection against dephosphorylation by AMP or A-769662 (Supplementary Fig. 1). We recently solved two different crystal forms of AMPK constructs that include the CBM but in neither case was there electron density present for this domain. Given that the CBM is required to achieve protection against dephosphorylation by small molecule activators, we reasoned that the presence of one of these compounds might stabilise the interaction between the CBM and the rest of the complex. Initial attempts at co-crystallisation with A-769662 were unsuccessful likely because of the relatively weak binding, poor solubility and the presence of additional, non-specific, binding sites that promote protein aggregation. We therefore looked for an alternative compound for co-crystallisation. From structures deposited in patent databases^40^, we selected and synthesised a cyclic benzimidazole derivative developed by Merck Sharp and Dohme Corporation and Metabasis Therapeutics , hereafter referred to as compound 991 (Fig. 1a and Supplementary Fig. 2). We found that 991 was 5- to 10-fold more potent than A-769662 in assays monitoring allosteric activation and protection against dephosphorylation (Fig. 1b and Table 1). We then determined the effectiveness of 991 in cell-based assays and found that it showed activation at much lower concentrations than required for A-769662 (Fig. 1c), in keeping with the enhanced dose response seen *in vitro*. Consistent with our previous studies with A-769662 (ref. 42), we found that 991 did not activate a complex lacking the CBM and that the dose response curve was shifted to the right upon mutation of Ser-108 (Fig. 1d).

**Figure 2:**
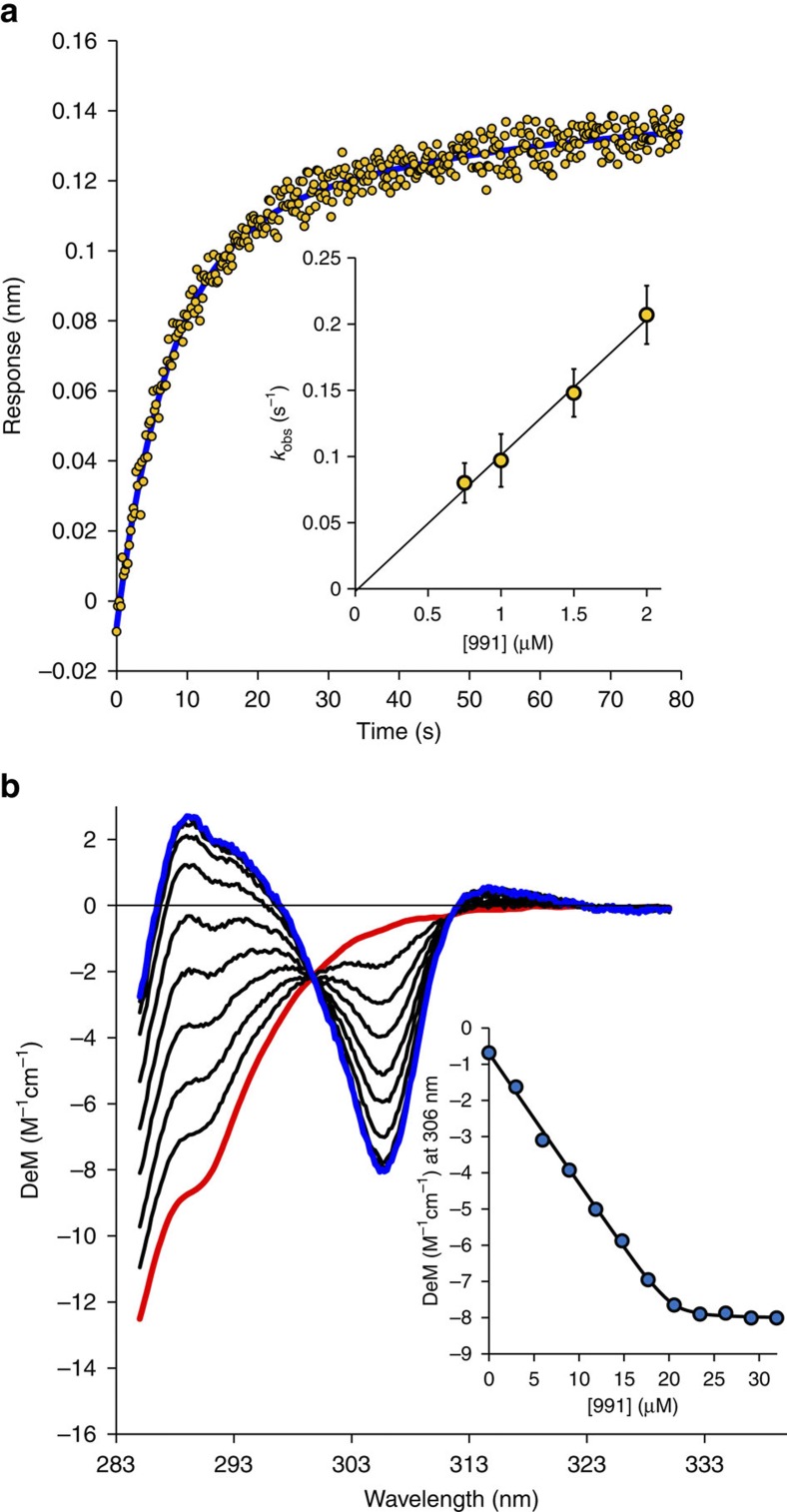
991 activator binding to AMPK. (**a**) BLI data (average of four traces) for the binding of 991 (1.5 μM) to α1β1γ1. A double exponential fit (blue) gave *k*_obs_=0.148 s^−1^. The dependence of *k*_obs_ on the concentration of 991 (inset) gave a *k*_on_ value of 0.103±0.008 μM^−1^s^−1^ (average of 4 traces). Analysis of the dissociation phase (see Supplementary Methods) gave *k*_off_=0.0062±0.0012, s^−1^, giving a *K*_d_ of 0.06±0.012 μM for the binding of 991 to α1β1γ1. (**b**) CD titration of 20 μM α1β1γ1 with 991 (0–32 μM). The spectrum of the protein (red) is very different from the spectrum of AMPK:991 complex (blue). Analysing the signal change at 306 nm as a function of 991 concentration (inset—1:1 binding model) gave a *K*_d_ of 0.078±0.026 μM.

Next, we compared binding of 991 and A-769662 to AMPK complexes using Biolayer Interferometry (BLI) and circular dichroism (CD) assays (see Fig. 2, Table 2 and Supplementary Methods). Several conclusions emerged:

1. Both the CBM and the kinase domain are required for effective compound binding.
2. AMPK complexes containing the β1 subunit bind the activators about 10 times stronger than β2 subunits (perhaps explaining the weaker activation displayed towards β2-containing complexes^43^ (Table 1)).
3. Compound 991 binds about 10-fold tighter than A-769662.
4. The activator dose–response curves monitoring AMPK activity, both in allostery and protection against dephosphorylation formats (Fig. 1), show the same patterns as the binding studies (Tables 1 and 2).

The CD data also establish that there is a single tight site for 991 binding whose structure we describe in the next section. While there was no CD change upon binding of A-769662, this compound competed for the binding of 991 to AMPK. Together, these data establish that there is a single-binding site common to A-769662 and 991, and likely related compounds^40^. The site requires both the CBM and kinase domain, and is responsible for both the allosteric activation and protection against dephosphorylation by small molecule activators but not by nucleotides. Interestingly, activation of AMPK by salicylate has also been shown to be ablated by removal of the CBM, or the mutation of Ser-108, implying that it too binds at the same site^48^.

### Structure of AMPK/activator complex

**Figure 3:**
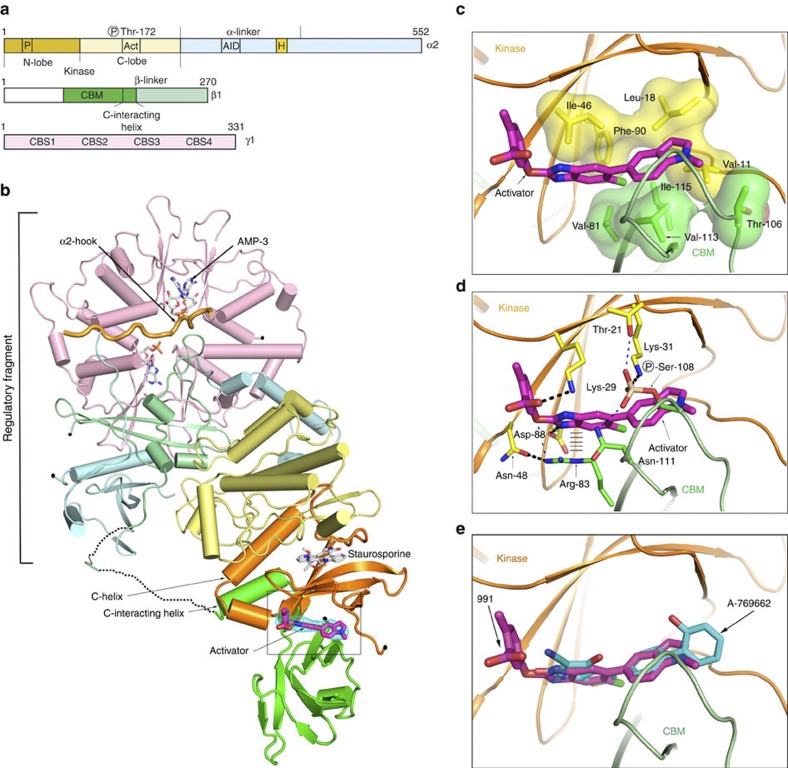
Structure of full-length AMPK complex with activator. (**a**) Bar diagram indicating the three subunits that make up the complex. (**b**) Cartoon representation of full-length α2β1γ1 in complex with the activator 991, the domains of the three subunits are coloured according to (**a**). The orientation of the figure is similar to our earlier paper, so that the kinase domain is ‘upside-down’ with respect to the classical kinase orientation. The activator, which binds at the interface of the kinase and CBM, is shown in stick representation with its carbon atoms coloured magenta. Omit density (Fo-Fc) covering the 991 compound is contoured at 2.5 sigma and coloured blue (see also Supplementary Fig. 3). (**c**) Detailed view of 991 binding in a pocket generated at the interface between the CBM and the kinase domain, and making interactions with a cluster of hydrophobic residues from each domain; Ile-46_(Kinase)_, Phe-90_(Kinase)_, Leu-18_(Kinase)_ and Val-11_(Kinase)_ and Val-81_(CBM)_, Val-113_(CBM)_, Ile-115_(CBM)_ and one of the side-chain carbon atoms (CG) of Thr-106_(CBM)_. These hydrophobic interactions mainly involve ring-1 and ring-2 of the activator (Fig. 1a). The hydrophobic residues from the kinase (yellow) and CBM (green) are shown as sticks with surfaces, while (**d**) shows the same view and details the polar interactions that contribute to activator binding. Ring-3, and its linkage to ring-2, are involved in a number of polar interactions. Salt-bridges are formed between Asp-88_(Kinase)_ and N2 of ring-2 and Lys-29_(Kinase)_ with the carboxyl group of ring-3. Lys-31_(Kinase)_ interacts with the phosphorylated serine (pSer) at position 108 from the CBM. The CBM contributes an important interaction to drug binding through Arg-83. In addition to making a hydrogen bond with Asn-48_(Kinase)_, it also makes a cation–π stacking interaction with ring-2 of the activator. The cation–π interaction from Arg-83_(CBM)_ with the activator is indicated by hatched lines. Additional potential interactions (where the bond lengths are longer than those expected for a high resolution structure) are indicated by a thin dashed blue line. (**e**) Overlay of A-769662 (cyan) and 991 (magenta) are shown in stick representation.

We solved the structure of full-length human α2β1γ1 AMPK bound to compound 991, and crystallographic statistics are presented in Table 3. The overall structure is shown in cartoon representation in Fig. 3a,b. The organization of the kinase domain with the regulatory complex (comprising the C-terminal domains of the α- and β-subunits, together with the γ subunit) is similar to our earlier report of the structure of the ΔCBM construct^38^ (the main-chain r.m.s.d. is 5.9 Å). In our new structure, the CBM is ordered and bound to the N-terminal lobe of the kinase domain. The interface between these two domains generates the binding cavity for 991 (Fig. 3 and Supplementary Fig. 3).

One end of the five stranded β-sheet that constitutes the core of the N-terminal domain of the kinase packs against a pair of anti-parallel β-strands from the CBM. 991 sits in a pocket generated at this interface and interacts with hydrophobic residues from each domain, mainly involving ring-1 and ring-2 of the activator (Figs 1a and 3c). Interestingly, the phosphorylated serine (pSer) at position 108 from the CBM is involved in a network of electrostatic interactions with Lys-31_(Kinase)_ and potentially Thr-21_(Kinase)_ and Asn-111_(CBM)_ (Fig. 3d). The role of pSer-108_(CBM)_ at the domain interface corroborates our earlier findings that this residue is important for the regulation and binding of compounds to AMPK^42^. We carried out site-directed mutagenesis experiments to test the importance of some of these polar interactions (Tables 1 and 2). Mutations of Lys-29_(Kinase)_/Lys-31_(Kinase)_, Arg-83_(CBM)_ and Ser-108_(CBM)_ all lead to more than a 25-fold reduction in activator affinity, without altering the regulation of AMPK by AMP (Table 1).

Utilizing the crystallization conditions for 991, we were able to refine conditions that gave crystals of AMPK in complex with A-769662. Although the diffraction data were limited to 3.9 Å, unbiased electron density maps (Supplementary Fig. 4) clearly show that A-769662 is located in the same binding site as 991 and how it overlaps with ring-1 and ring-2 of 991 (Fig. 3e). The two activator complex structures therefore confirm the interpretation of our functional and binding data that A-769662 and 991 activate AMPK through binding to the same site at the interface of the kinase domain and CBM.

An α-helix immediately C-terminal to the CBM, absent in previous crystal structures of the CBM^38^ and just outside of the CBM consensus sequence, interacts with the αC helix of the kinase (hereafter, the C-interacting helix) (Supplementary Fig. 5). The two helices are oriented approximately antiparallel with three residues at the N-terminus of the latter (Val-162, Phe-163 and Leu-166) making hydrophobic contacts with the αC helix. Sequence alignment of this region demonstrates that the C-interacting helix is conserved in AMPKs across numerous species (Supplementary Fig. 6; note, there is a high sequence identity of the entire β1 sequence across all species (over 95%)). We designed a mutation in the C-interacting helix with the aim of blocking its interaction with αC (Leu-166->Glu). The resulting AMPK complex showed a reduction in allosteric activation by 991 while, importantly, allosteric activation by AMP was not affected (Supplementary Fig. 7). The role of the C-interacting helix in mediating allosteric activation by 991 has close parallels in other kinases^49^. For example, cyclin-dependent kinases (CDKs) are allosterically activated by binding of their regulators, the cyclins. An alpha helix from the cyclin packs against the αC helix of the CDK kinase domain promoting an active conformation (Supplementary Fig. 8).

Most structural features of the activator complex of AMPK presented here show the hallmarks of an activated kinase but one feature of the kinase domain does not (a detailed comparison of the CBM/activator/kinase structure with our earlier ΔCBM structure is presented in Supplementary Figs 9–12). In brief, the residues that contribute to the regulatory and catalytic spines are all in place, similar to our earlier structure, consistent with an active kinase structure. Similarly, the activation loop is fully ordered and continues to mediate the interaction of the kinase with the regulatory fragment. However, the top of the C-helix is rotated away from the active site, relative to our earlier structure, such that the Lys-45->Glu-64 interaction is broken (which is regarded as essential for efficient phosphoryl transfer). This interaction is still intact in the CDK–cyclin complex despite the similar structural packing of the CBM and cyclin against the αC helices of AMPKα and CDK, respectively. Importantly, we are able to activate AMPK in cells and in cell-free assays by 991 and A-769662, suggesting that this Lys-Glu interaction does form during the catalytic cycle. This ‘swung out’ αC conformation has been widely seen in other systems^50^, and it has been argued that the multiple observations of this conformation suggest that it highlights conformational changes required for an active kinase. Specifically, since ADP release is considered to be the rate-limiting step in the catalytic cycle, and not phosphoryl transfer, it is thought that a conformational flip in the DFG motif is necessary for this slow step and that movement of the C-helix facilitates this. Thus, by analogy with other kinases the conformation captured in our activator complex, in the absence of ATP and substrate, suggests that facilitation of movement in the αC-helix may be an important contribution to activation of AMPK.

### α2-hook and the autoinhibitory domain (AID)

**Figure 4:**
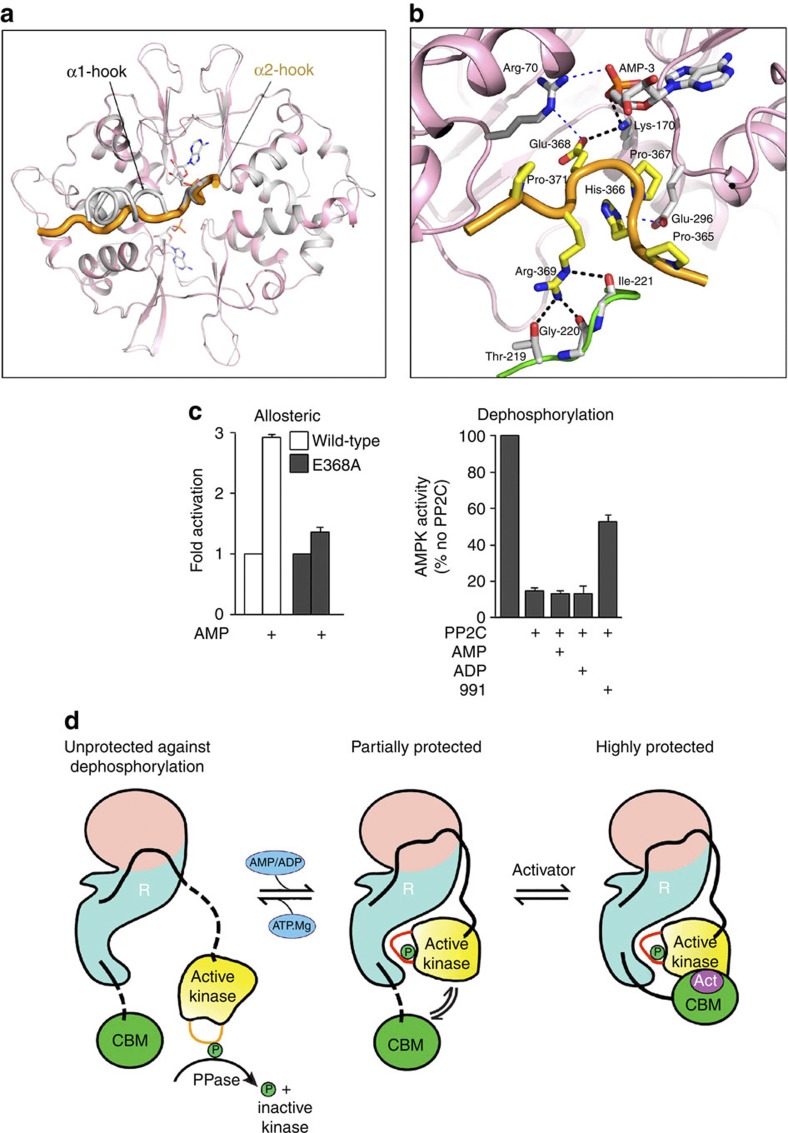
The structure of the α2 hook of AMPK. (**a**) Ribbons representation of the overlap of the γ subunit and hook regions from full-length α2/activator complex (coloured in pink and orange) and from the ΔCBM AMPK complex (coloured in grey), showing the close structural similarity of the hook region for α1 and α2 over the five strictly conserved residues. The two exchangeable AMP moieties are shown in stick representation (from the full-length structure) but the non-exchangeable AMP has been removed for clarity. (**b**) Detailed view of the α2 hook (coloured in orange with the residue carbons in yellow) and its interaction with residues at the AMP-3 site on the γ-subunit (coloured pink with the residue carbons in grey) and with a loop from the β-subunit (coloured green with the carbons in grey). AMP-3 is shown in stick representation (with the carbons coloured in grey). His-366_(α2-hook)_ forms a salt bridge with Glu-296_(γ)_, while the following proline at position 367 introduces a kink into the chain, which seems instrumental in positioning Glu-368_(α2-hook)_ that makes salt-bridges to Lys-170_(γ)_ and Arg-70_(γ)_. Arg-369_(α2-hook)_ makes hydrogen bonds with three residues on a loop from the β-subunit, Thr-219_(β)_, and the main-chain carbonyl oxygen of Gly-220_(β)_. Potential interactions are indicated by a thin dashed blue line. (**c**) Allosteric activation of wild-type and E368A_(α2-hook)_ α2β1γ1 complex by AMP (100 μM) (Left). In the right-hand panel, the effect of AMP (50 μM), ADP (50 μM) or 991 (100 nM) on protection against dephosphorylation for the E368A_(α2-hook)_ α2β1γ1 complex (Right). Results are the mean ±s.e.m. for three independent experiments. (**d**) Schematic for the regulation of AMPK by kinase domain and CBM tethering to the regulatory fragment. The right-hand panel represents the activated full-length AMPK/991 complex reported in this paper. It consists of the regulatory fragment (R) containing the γ-subunit (in pink) and the C-termini scaffold domains of the α- and β-subunits (in light blue/green). The α2 kinase domain (phosphorylated on Thr-172 of the activation loop) is shown in yellow and is connected to the regulatory fragment by a flexible linker (in black). The interaction of the kinase domain with the regulatory fragment mainly involves the activation loop and protects Thr-172 from dephosphorylation. The CBM of the β-subunit (coloured in green) binds to the N-lobe of the kinase domain and is also connected to the regulatory fragment by a flexible linker (in black). The presence of the activator compound 991 (Act) is envisaged to strengthen the interaction between the kinase and CBM and protect a major proportion of the active enzyme against dephosphorylation. Dissociation of the activator compound gives rise to the species shown in the middle panel. In this case the enzyme becomes less active because the interaction between the CBM and the kinase domain is weaker and they therefore interact for a smaller proportion of the time. Replacing ADP (or AMP) by Mg.ATP leads to displacement of the α-hook and thus the dissociation of the kinase domain and CBM from the regulatory fragment (as shown in the left panel). In this form, the kinase is no longer allosterically activated and is susceptible to dephosphorylation, and thus inactivation.

The complex used in this study contains the α2 isoform, compared with α1 in our previous structure^38^, and in both cases there is a segment in the α-subunit, between the N-terminal kinase domain and the C-terminal scaffolding domain, that interacts with one of the exchangeable nucleotide-binding sites (Site-3) on the γ-subunit (Fig. 4). In the current structure, we term this α2-hook residues (defined as residues α2 365–371 and referred to as α-RIM in Chen *et al*.^51^), and it is very similar for that seen for α1, suggesting that the hook plays a similar role in communicating the nucleotide status at AXP Site-3 (see below). The improved resolution of the X-ray data used in the present study makes the identification of the sequence register in the α2-hook region unambiguous, and it is different from the sequence register we built into our earlier α1 structure. Wu and colleagues^51^ have recently suggested an alternative way of building the hook sequence in our earlier electron density maps for the α1 complex and we agree with their reinterpretation of the sequence register (α1, 359–365; α2 365–371). We have reviewed our previous structure of α1 in the light of this and the current structure and found some additional areas that can now be interpreted more fully than was possible before (Supplementary Figs 13–15) and have deposited revised coordinates at the Protein Databank (PDB ID: 4CFH).

Comparison of the α1 and α2 structures shows that the hook regions make the same interactions over the five residues that are conserved between them (Pro-His-Pro-Glu-Arg) (Fig. 4b). Interestingly, Glu-368_(α2-hook)_ makes salt-bridges with Lys-170_(γ)_ and potentially Arg-70_(γ)_ (Fig. 4b), and the latter residue we have argued mediates the initial signal that distinguishes between AMP/ADP and ATP being bound at the exchangeable adenine nucleotide Site-3 (ref. 38). Mutation of Glu-368_(α2-hook)_ ablates nucleotide protection against dephosphorylation (Fig. 4c) consistent with our proposal that site-3 mediates this effect^38^. We also find that this mutation reduces allosteric activation by AMP (Fig. 4c) as reported previously^51^. How this mutation affects both aspects of nucleotide regulation is discussed below.

Chen *et al*.^51^ have also suggested how their isolated AID (autoinhibitory domain) crystal structure can be docked onto our earlier ΔCBM structure. In our current structure there is significantly better density for the α3 helix of the AID where the sequence register is now convincing, and reasonable main chain, but not side-chain, definition for α1 helix (Supplementary Fig. 14). Again there is not much electron density for other parts of the domain, but our new structure further supports the interpretation of Chen *et al*.^51^ that the α1 and α3 helices of the AID adopt a similar structure in the isolated domain and in full-length AMPK. Comparison of our structures with the yeast kinase/AID structure (PDB ID: 3H4J) shows that the AID has undergone a major rigid-body rotation such that it mostly interacts with the regulatory fragment and not the kinase. What does this mean for the autoinhibitory function? The AID can exist in a kinase-bound state and in a regulatory fragment-bound state. The former is understood to be inhibitory and the latter active. Given its location between the kinase domain and the α-hook, it is plausible that the switch in position of the AID occurs in response to which AXP is bound to the regulatory fragment, providing a signalling mechanism for nucleotide regulation^52^.

### Protection against dephosphorylation

In our previous paper^38^, we suggested that AMPK is protected against dephosphorylation by AMP/ADP binding to the γ-subunit and stabilizing the interaction of the α-hook, and thus enhancing the recruitment of the kinase domain to the AMPK regulatory fragment. In the current structure, as in the ΔCBM structure, nearly all the contacts with the regulatory fragment are made by residues on the activation loop of the kinase, thus stabilizing the activation loop structure. While the phosphate group of Thr-172 is partially solvent exposed, it is not accessible to the active site of a phosphatase. To be dephosphorylated, the activation loop must undergo a conformational change that enables the phosphate of pThr-172 to be flipped out of the RD pocket of the kinase. The interactions of the regulatory fragment of AMPK with the activation loop thus leads to protection of pThr-172 (refs 1, 2, 38).

In our current structure, the CBM is recruited to the core complex by interaction with the kinase domain (Figs 3 and 4d). The interaction of the CBM with the kinase domain similarly makes it less likely that the kinase domain will dissociate from the regulatory fragment. Thus, by promoting this CBM/Kinase/regulatory–fragment interaction, 991 and A-769662 enhance protection of the activation loop against dephosphorylation. As mentioned above (and in Supplementary Fig. 1), protection against dephosphorylation by AMP requires the CBM thus implying that the CBM/kinase interface described here is important for protection against dephosphorylation by physiological regulators. The fact that nucleotide-mediated protection against dephosphorylation is a weaker effect than that shown by compounds is consistent with the interaction between CBM/kinase being weaker in the absence of 991.

As described above, mutation of Glu-368_(α2-hook)_ reduces the extent of allosteric activation, as well as protection against dephosphorylation. Does this imply that is site-3 responsible for both aspects of activation? We previously presented data that demonstrated that the AMP dose dependence of the allosteric effect was consistent with it being mediated by binding to the tighter nucleotide binding site (site-1) while the protection effect had a weaker dose response consistent with site-3. These observations are reconciled on the assumption that allosteric activation by AMP occurs when the kinase is bound to the regulatory fragment. Indeed, it is difficult to envisage how conformational changes in the regulatory fragment could be propagated to the kinase domain unless it is bound to the regulatory fragment. Therefore, mutations that reduce the proportion of AMPK molecules with ‘bound kinase’, like Glu-368->Ala, should reduce the extent to which AMP can activate.

## Discussion

Our current structure and binding studies reveal the site on AMPK that mediates the activation by compounds, including A-769662, 991 and perhaps salicylate. Inspection of the CBM/kinase interface, with the activator removed (Supplementary Fig. 16), reveals that the protein subunits make contacts around the rim of the interface but leave a large, significantly hydrophobic, cavity where the activator binds. It is tempting to speculate, therefore, that this cavity has evolved to bind a natural ligand that acts to stimulate AMPK. Although we have not yet managed to identify this ligand we think that it is likely to exist and that it represents an important missing link in our understanding of the physiological regulation of AMPK.

## Methods

### Materials

The following antibodies were used in this study. The source and catalogue number are indicated in parentheses. anti-GFP (Clontech, Catalogue number 632381), anti-pACC (Cell Signalling, Catalogue number 3661), anti-pT172 (Cell signalling Catalogue number 2535), anti-AMPKβ1/β2 (Cell signalling Catalogue number 4150), Goat anti-rabbit HRP (ProteinSimple, Catalogue number 71598) and Goat anti-mouse IRDye 800CW (Li-Cor, Catalogue number 926-32210).

### Constructs

Human AMPK subunits (His-α1/2, β1/2 and γ1) were cloned into a pET-3d vector, and successive rounds of cloning were performed to generate a tricistronic vector similar to that previously reported (Neumann *et al*.,^53^). For BLI and crystallization experiments an AVI-tag (GLNDIFEAQKIEWHE) was engineered onto the N-terminus of the β-subunit and cloned into a pET-3d vector. The tricistronic vector was generated as previously reported (Neumann *et al*.,^53^). Biotin ligase (BirA) lacking a His-tag was amplified from His-BirA pET-47 vector (a kind gift from Vangelis Christodoulou) and cloned into pET-47 vector.

### AMPK purification

Recombinant AMPK complexes were expressed in *E. coli* BL21 cells (BL21-one shot DNA Star, Stratagene) and purified by nickel affinity chromatography (His-Trap, GE Healthcare) and gel filtration (Superdex 200, GE Healthcare). AMPK complexes were phosphorylated by incubation with CaMKKβ in the presence of 0.5 mM ATP, 2.5 mM MgCl_2_ and 0.5 mM AMP overnight at 18 °C as previously reported^38^. The phosphorylated AMPK complex was repurified by Nickel-sepharose and gel filtration. For the BLI experiments, the AVI-tagged AMPK complexes were coexpressed with a biotin ligase (BirA) to generate biotinylated AMPK that could be purified (as above) and immobilized onto superstreptavidin sensors on an Octet RED biolayer interferometer. The growth media for expressing the biotinylated complexes was supplemented with 50 μM D-Biotin. It was discovered later in this study that the endogenous bacterial BirA produced sufficient biotinylated AMPK complex without overexpressing the BirA in bacteria and supplementing the media with biotin.

### AMPK functional assays

For allosteric effects, AMPK activity was determined by phosphorylation of the SAMS peptide in the presence or absence of varying concentrations of compound, as indicated in the appropriate figures. Results are plotted as fold activation relative to the activity in the absence of added compound and are the mean±s.e.m. from at least three independent experiments. For dephosphorylation studies, an aliquot of phosphorylated AMPK was incubated in 50 mM HEPES, pH 7.4, 100 mM NaCl, 2.5 mM MgCl_2_, in the presence or absence of recombinant PP2Cα (26 ng) and in the presence or absence of varying concentrations of compound for 20 min at 37 °C (or as indicated in the appropriate figure legends). For determination of AMPK activity, the reaction mixture was diluted 1:25 in 50 mM Hepes, pH 7.4 to prevent further dephosphorylation. The appropriate compounds were added to the diluted samples in order to equalize their final concentration in all samples and AMPK activity measured using the SAMS peptide assay. Results are plotted as a percentage of the activity measured in the absence of PP2C and are the mean±s.e.m. from at least three independent experiments.

### Endogenous AMPK activity in HEK293 cells

HEK293 cells (from American Tissue Culture Collection (ATCC)) were grown at 37 °C with 5% CO_2_ in Dulbecco’s modified Eagle’s medium (4,500 mg l^−1^ glucose with sodium pyruvate and pyridoxine) supplemented with 10% fetal calf serum and 1 mM glutamine^42^. Cells were transferred into serum-free media for 2 h before treatment with varying concentrations of A-769662 or 991, as indicated in the figure legends. Cells were washed briefly with ice-cold phosphate-buffered saline before rapid lysis in ice-cold buffer (50 mM HEPES, pH 7.4, containing protease inhibitor mixture (Roche Applied Science), 1 mM EDTA, 10% glycerol (v/v), 50 mM NaF and 1% (v/v) Triton X-100). Insoluble material was removed by centrifugation at 10,000 *g* for 10 min at 4 °C, and the supernatant was used for subsequent analysis.

### Transient transfection of HEK293 cells

Plasmid DNA was prepared using a Qiagen maxiprep kit according to the manufacturer’s instructions. Cells were transfected by Ca^2+^-phosphate precipitation with 10 μg of each plasmid encoding myc-α1 and FLAG-γ1 subunits, and either wild-type β1, β1 harbouring the S108A mutation or lacking the CBM (ΔCBM; β1, 186–270)^42^. Thirty hours post transfection, cells were collected as described above. cDNA encoding the β1 subunit was cloned into a vector to allow expression of a fusion protein with green fluorescent protein (N-terminal of β1). This allowed us to determine the expression of the β1 subunit using western blot analysis. The primary antibody was used at 1:1,000 dilution, and the secondary antibody was used at 1:10,000 dilution.

### Immunoprecipitation of AMPK from mammalian cells

Endogenous AMPK was immunoprecipitated using a rabbit antipan-β antibody^42^ (1:10,000 dilution). Recombinant AMPK from transfected cells was immunoprecipitated using an anti-FLAG antibody. Immune complexes were washed extensively, and AMPK activity was determined by performing an SAMS assay.

### Simon western blot analysis (ProteinSimple, California, USA)

In some cases, western blot analysis was performed using a capillary-based automated system (http://www.proteinsimple.com/simon.html). The western blot was performed using the standard manufacturer’s protocol using a primary antibody dilution of 1:200 and the secondary antibodies from ProteinSimple were used neat.

### Biolayer interferometry

The binding of A-769662 and A-769662-like compounds to different AMPK constructs was measured on an Octet RED biolayer interferometer (Pall ForteBio Corp., Menlo Park, CA, USA). AMPK constructs biotinylated by addition of an AviTag sequence to the N-terminus of the β subunit were immobilized on superstreptavidin (SSA) biosensors (Pall ForteBio Corp., Menlo Park, CA, USA) at a concentration of 40 μg ml^−1^. Binding of compounds at concentrations in the range 0.75 to 10 μM was measured at 25 °C using 1–5 min association steps and 1–10 min dissociation steps. The buffer was Fortebio kinetics buffer (10 mM phosphate (pH 7.4), 150 mM NaCl, 0.1 mg ml^−1^ BSA and 0.005% Tween-20) supplemented with 1 mM TCEP and a final DMSO concentration of 2%. In the majority of cases, some non-specific binding and/or instrument drift meant that the association and dissociation phases could not be fit to a single-exponential function. The single observed rate (*k*_OBS_) employed in the analyses was the one that accounted for at least 85% of the total observed reaction amplitude (Supplementary Methods). Kinetic constants (*k*_on_ and *k*_off_) were obtained from plots of the association phase *k*_OBS_ values versus compound concentration. In some cases an accurate value for *k*_off_ could not be determined from such plots, and in these cases this constant was determined independently from analysis of the dissociation phase (see Supplementary Information).

### Circular dichroism

Near-UV CD spectra (340–255 nm) were recorded on a Jasco J-815 spectropolarimeter fitted with a cuvette holder thermostatted by a CDF-426S/15 Peltier unit. All measurements were made at 20 °C in 10-mm path length fused silica cuvettes (Hellma, Jena, Germany). Spectra were typically recorded with 0.1-nm resolution and baseline corrected by subtraction of the appropriate buffer spectrum (50 mM Tris, pH 8.0, 300 mM NaCl and 1 mM TCEP). Molar CD extinction coefficients (Δ*ε*_M_) were calculated by dividing with the protein concentration.

### Crystallography

Full-length AMPK, γ1(human, 1–331) AVI-tag-β1(human, 1-270) His-α2(human, 1–552), was cloned into a tricistronic vector and subsequently expressed in *E. coli* BL21 Star (DE3). Proteins were purified using a nickel affinity chromatography and gel filtration. The purified protein was phosphorylated using CAMKKβ kinase overnight at 18 °C then repurified by Nickel affinity chromatography and gel filtration. The complex stock solution was prepared at 7 mg ml^−1^ in 50 mM Tris, pH 8.0, 300 mM NaCl and 1 mM TCEP, mixed with a threefold molar excess of AMP and onefold of staurosporine and 991 compound. Crystals were grown by vapour diffusion technique at 4 °C in hanging drops. Drops were prepared by mixing equal volumes of protein complex with reservoir solution containing 13% PEG3350, 0.1 M MgCl_2_, 1% Glucose, 0.15% CAPB in 100 mM Imidazole (pH 6.2). Crystals were first transferred into mother liquor with an additional 30% ethylene glycol, before plunging into liquid nitrogen. Diffraction data were collected on a Pilatus 2 M detector (Dectris), Diamond Light Source, Oxford. Data were integrated using Denzo and scaled with Scalepack^54^. The structure was solved by molecular replacement using Phaser^55^ and standard refinement was carried out with Phenix^56^ using 2Y94.pdb and 2F15.pdb as the search models, with manual model building with COOT^57^. General crystallographic calculations were carried out using the CCP4 package^58^. Figures were created with Pymol (http://pymol.sourceforge.net/).

## Author contributions

B.X., M.J.S., D. Carmena, N.J.B., L.F.H., E.U., B.R.P., R.B.H., P.A.W., S.H., F.G., S.R.M., and D. Carling performed experiments. All authors contributed to data analysis, experimental design and manuscript writing.

## Additional information

**How to cite this article:** Xiao, B. *et al*. Structural basis of AMPK regulation by small molecule activators. *Nat. Commun.* 4:3017 doi: 10.1038/ncomms4017 (2013).

**Accession codes:** Atomic coordinates and structure factors have been deposited in the Protein Data Bank under accession codes 4CFE (AMPK:991 complex), 4CFF (AMPK:A-769662 complex) and 4CFH (AMPK ΔCBM, revised co-ordinates).

## Supplementary Material

Supplementary Information Supplementary Figures 1-22, Supplementary Table 1, Supplementary Methods and Supplementary Reference

## Figures and Tables

**Table 1:** Allosteric activation of AMPK complexes by 991 and A-769662.

**Complex**	**Activation (fold)**			***A***_**0.5**_ **(μM)**	
	**991**	**A-769662**	**AMP**	**991**	**A-769662**
α1β1γ1	4.8 (0.1)	2.0 (0.1)	1.5 (0.1)	0.03 (0.01)	0.59 (0.07)
α1β2γ1	2.4 (0.1)	No activation	1.4 (0.1)	1.1 (0.3)	ND
α2β1γ1	12.2 (0.6)	14.3 (0.4)	2.6 (0.1)	0.09 (0.02)	0.39 (0.03)
α2β2γ1	5.4 (0.4)	No activation	3.0 (0.2)	0.51 (0.19)	ND
ΔCBM	No activation	No activation	2.2 (0.1)	ND	ND
α2_(K29/K31)_β1γ1	4.0 (0.6)	No activation	1.5 (0.1)	2.3 (0.8)	ND
α2β1_(S108A)_γ1	11.6 (2.0)	No activation	2.1 (0.1)	3.6 (1.2)	ND
α1β1_(R83A)_γ1	No activation	No activation	1.4 (0.1)	ND	ND
α2β1_(L166E)_γ1	2.0 (0.1)	No activation	1.8 (0.1)	0.12 (0.04)	ND

AMPK activity was measured using the SAMS peptide assay. Allosteric activation by 0.1 mM AMP is also shown. In all cases, results shown are the mean (±s.e.m.) determined from at least three independent experiments. In some cases, no activation by compound was detectable up to concentrations of 10 μM, and in these cases the *A*_0.5_ was not determined (ND). Concentrations of 991 or A-769662 above 10 μM inhibited AMPK possibly through a deleterious effect on the enzyme. The specific activities of the various AMPK complexes (following phosphorylation by CaMKKβ) were similar, and, in the absence of 991, A-769662 or AMP was within a range varying between 300 and 500 nmol^−1^min^−1^mg^−1^. The activity of the α2β1_(R83A)_γ1 complex was too low to measure accurately and so is not included here. As a result the α1β1_(R83A)_γ1 activity data are included. ΔCBM, AMPK complex (α1β1_(185-270)_γ1) lacking the carbohydrate-binding module (CBM).

**Table 2:** Equilibrium *K*_d_ values for the binding of A-769662 and 991 compounds to phosphorylated AMPK.

	**BLI**		**CD**
**AMPK complex**	**A-769662 (μM)*****K***_**d**_	**991 (μM)*****K***_**d**_	**991 (μM)*****K***_**d**_
α1β1γ1	0.51 (0.14)	0.06 (0.012)	0.078 (0.03)
α2β1γ1	0.40 (0.15)	0.06 (0.013)	0.085 (0.03)
α1β2γ1	14.5 (5.1)	0.51 (0.19)	1.18 (0.31)
ΔCBM	17.9 (5.7)	>25	51 (11)
ΔKD	49 (12)	4.1 (0.9)	ND
α1_(K31A/K33A)_β1γ1	5.4 (1.3)	2.05 (0.38)	
α1_(R83A)_β1γ1	8.6 (2.2)	1.90 (0.53)	
α1β1_(S108A)_γ1	33.2 (3.5)	8.9 (2.8)	
α2_(K29A/K31A)_β1γ1	14.4 (4.1)	3.70 (0.50)	
α2β1_(R83A)_γ1	ND	1.57 (0.41)	

BLI, biolayer interferometry; CD, circular dichroism; ND, not determined.

Dissociation constants (*K*_d_) were determined using BLI or CD. The *K*_d_ values are reported as the mean (±s.d.) determined from at least 4 independent experiments.

ΔCBM, AMPK complex (α1β1_(185–270)_γ1) lacking the carbohydrate-binding module (CBM). ΔKD, AMPK complex (α1_(396–548)_β1γ1) lacking the kinase domain (KD).

All complexes were human except rat α1_(396–548)_ in the ΔKD complex. In addition, this protein is not phosphorylated since it lacks Thr-172.

**Table 3:** Data collection and refinement statistics (Molecular replacement).

	**α2AMPK complex with 991**	**α2AMPK complex with A-769662**
	**PDB ID: 4CFE**	**PDB ID: 4CFF**
*Data collection*		
Space group	P21	P21
*Cell dimensions*		
*a*, *b*, *c* (Å)	76.03, 134.14, 140.56	76.02, 134.79, 141.29
α, β, γ (°)	90, 92.42, 90	90.00, 93.04, 90.00
Resolution (Å)	30.0–3.02 (3.21–3.02)*	20.0–3.92 (4.14–3.92)*
*R*_sym_ or *R*_merge_	0.043 (0.44)	0.083 (0.33)
*I*/σ*I*	21.0 (2.2)	9.8 (2.2)
Completeness (%)	95.1 (93.1)	95.0 (82.6)
Redundancy	2.7 (2.7)	3.2 (2.8)

*Refinement*		
Resolution (Å)	19.91–3.02	19.93–3.92
No. reflections	50545	23906
*R*_work/_ *R*_free_	21.8/25.3	20.8/26.3
*No. atoms*		
Protein	14021	14270
Ligand/ion	270	214
Water	31	—
*B-factors*		
Protein	78.3	87.6
Ligand/ion	76.0	91.2
Water	44.7	—
*r.m.s. deviations*		
Bond lengths (Å)	0.002	0.006
Bond angles (°)	0.653	0.684
*Molprobity Statistics*		
Ramachandran	95% Favoured, 0.4% outliers	94% Favoured, 0.6% outliers

*Data collection*		
Wavelength	0.92 A	0.92 A
Temperature	100 K	100 K
Beamline	I04-1	I04-1

One crystal was used for the each structure above.

^*^Highest resolution shell is shown in parenthesis.
