# Supplementary material for: Structural basis of AMPK regulation by small molecule activators
Source: Nat Commun. 2013 Dec 19;4:3017. doi: 10.1038/ncomms4017 (PMC3905731; doi:10.1038/ncomms4017)
Supplement: Supplementary Information — Supplementary Figures 1-22, Supplementary Table 1, Supplementary Methods and Supplementary Reference [file ncomms4017-s1.pdf]

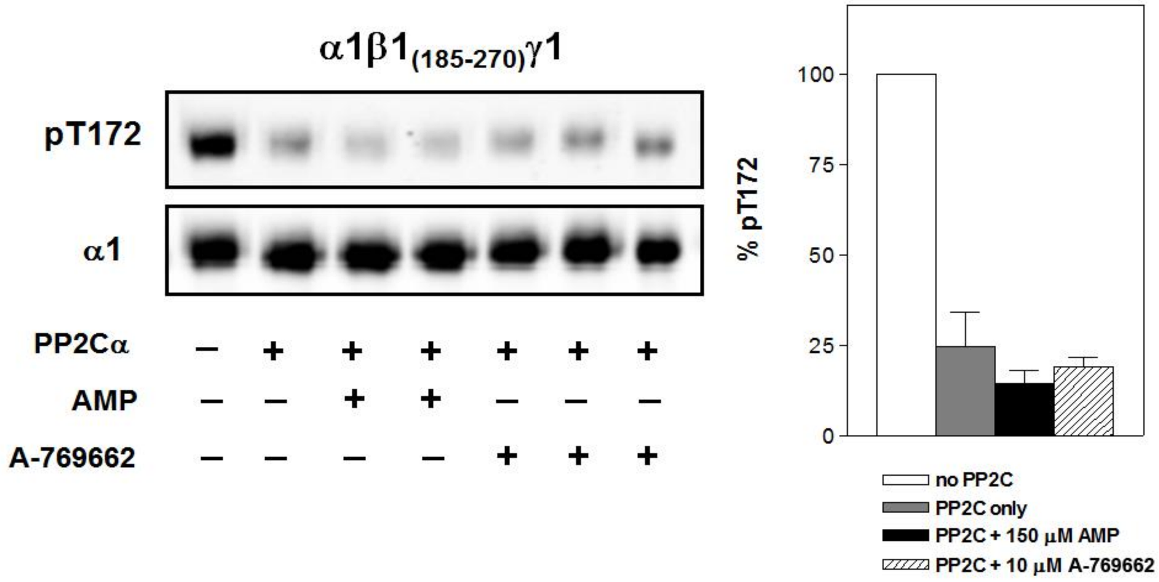

Supplementary Figure 1. AMP and A-769662 require the presence of the carbohydrate binding module (CBM) to protect pThr-172 from dephosphorylation.

AMPK complex lacking the CBM was purified by Nickel affinity chromatography and phosphorylated by incubation with CaMKKβ for 10 mins. An aliquot of phosphorylated AMPK was incubated in 50 mM HEPES, pH 7.4, 100 mM NaCl, 2.5 mM $MgCl_2$, in the presence or absence of recombinant PP2Cα (26 ng) and in the presence or absence of AMP (150 µM) or A-769662 (10 µM) for 20 min at 37°C. The dephosphorylation reaction was terminated by the addition of gel-loading buffer and samples were resolved by SDS-PAGE and subjected to Western blot analysis. Thr-172 phosphorylation was determined using rabbit anti-pThr-172 antibody (Cell Signaling). Total AMPK was detected using sheep anti-α1. Primary antibodies were detected using LI-COR IRDye® Infrared Dye secondary antibodies and visualised using an Odyssey Infrared Imager (LI-COR Biotechnology). Quantification of results was performed using Odyssey software and expressed as a ratio of the signal obtained with the phospho-specific antibody relative to the appropriate total antibody. The relative quantitation of pThr-172:total α is plotted as a percentage of the value in the absence of PP2C. A representative blot is shown and data displayed as the average ± S.E.M. from at least 3 independent experiments.

^{1}H-NMR spectrum:

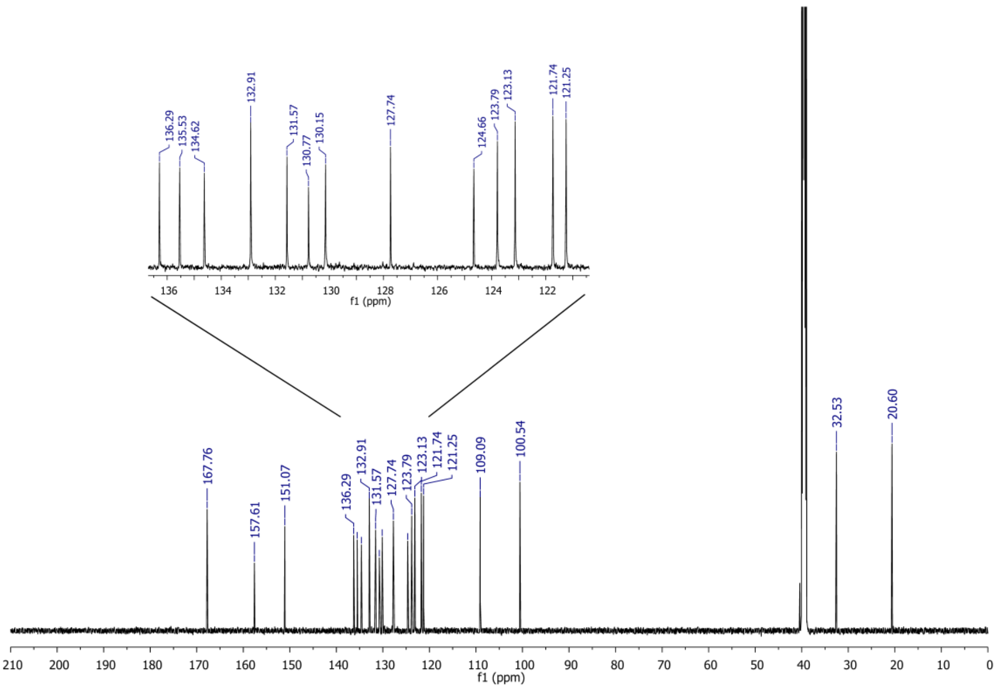

^{13}C-NMR spectrum:

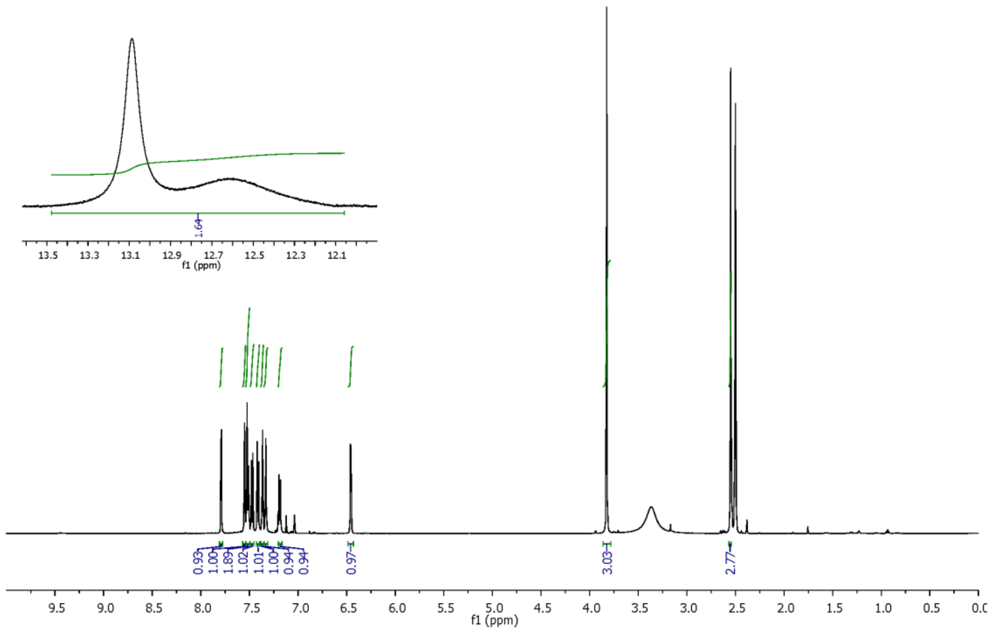

Supplementary Figure 2. Synthesis and characterization of 991 compound.

991 (5-{[6-chloro-5-(1-methyl-1H-indol-5-yl)-1H-benzimidazol-2-yl]oxy}-2-methylbenzoic acid) was synthesised following the procedure earlier described[59].

^{1}H-NMR (600 MHz, DMSO-d_6): δ 13.09 p.p.m. (s, br, 1H),12.61 (s, br, 1H), 7.79 (d, *J*=2.6 Hz, 1H), 7.55 (s, 1H), 7.53-7.50 (m, 2H), 7.47 (d, *J*=8.4 Hz, 1H),7.41 (d, *J*=8.4 Hz, 1H), 7.37 (d, *J*=2.9 Hz, 1H), 7.33 (s, 1H), 7.19 (d, *J*=8.4 Hz, 1H), 6.46 (d, *J*=2.9 Hz, 1H), 3.82 (s, 3H), 2.55 (s, 3H); ^{13}C-NMR (150 MHz, DMSO-d_6): δ 167.8 p.p.m., 157.6, 151.1, 136.3, 135.5, 134.6, 132.9, 131.6, 130.8, 130.2, 127.7, 124.7, 123.8, 123.1, 121.7, 121.2, 109.1, 100.5, 32.5, 20.6; HRMS (*m/z*): [M+H]+ calcd for $C_{24}H_{18}ClN_3O_3$, 432.1115; found, 432.1122.

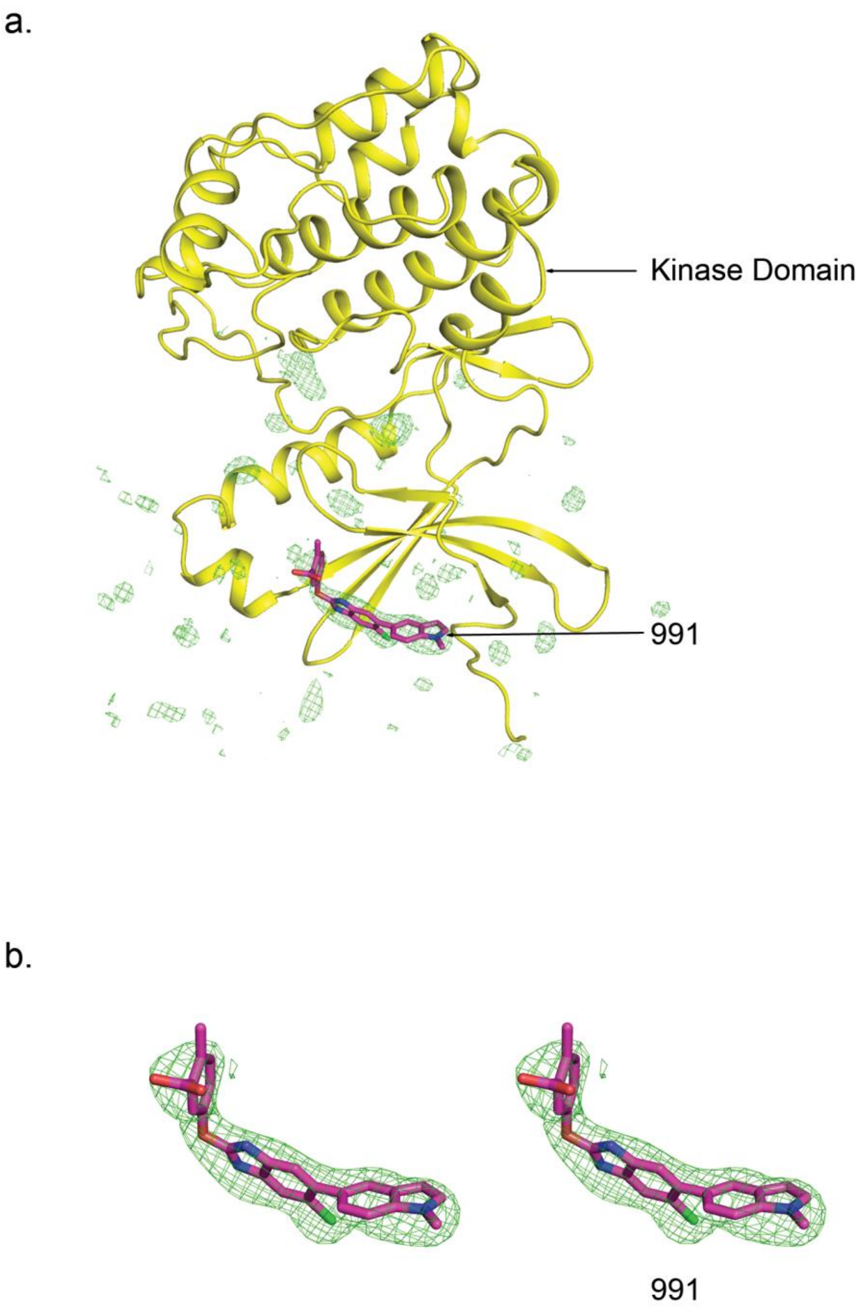

Supplementary Figure 3. Omit map for 991. a) α2 kinase domain in cartoon representation and 991 with an Fo-Fc omit map contoured at 3.0σ (cover radius of 20 Å). b) Stereo pair of the same omit electron density map at 2.0 σ with a cover radius of 2.5 Å.

a.

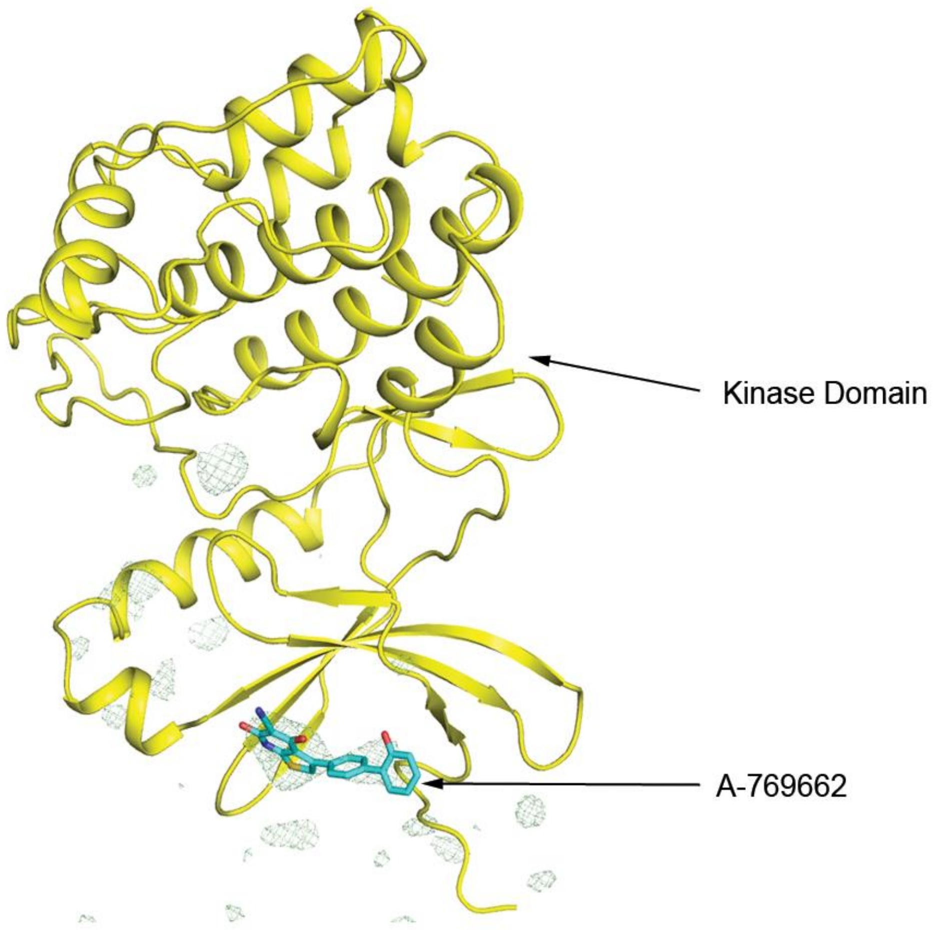

b.

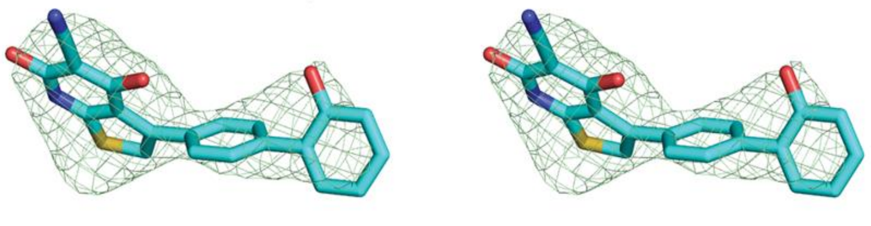

Supplementary Figure 4. Omit map for A-769662. a) α2 kinase domain in cartoon representation and A-769662 with an Fo-Fc omit map contoured at 3.0σ (cover radius of 20 Å). b) Stereo pair of the same omit electron density map at 2.0 σ with a cover radius of 2.5 Å.

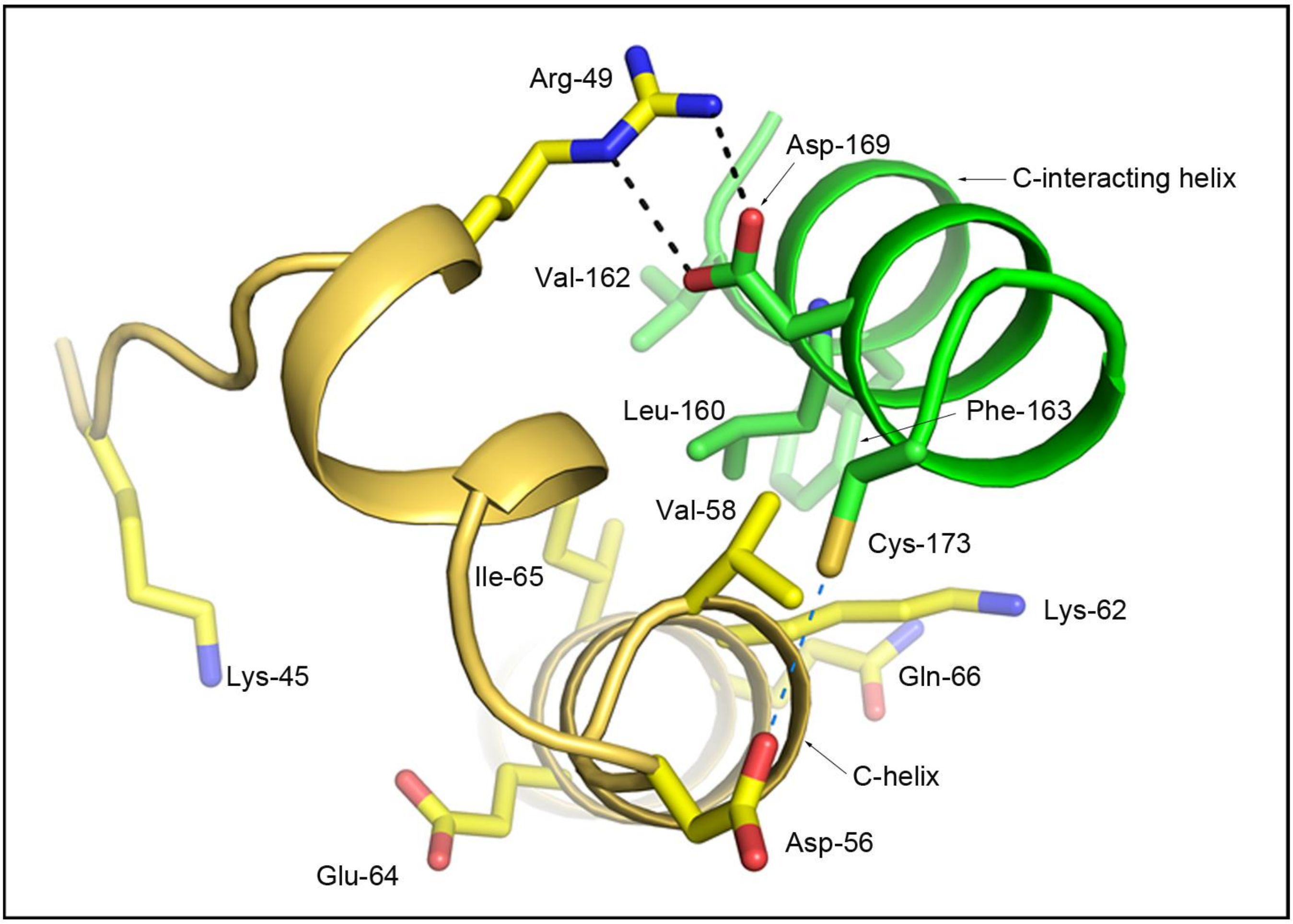

Supplementary Figure 5. Interactions between the C-interacting helix and the kinase domain αC helix. Ribbons representation of some of the interactions between the C-helix of the kinase (in yellow) and the C-interacting helix of the CBM (in green). There is an α-helix that extends from the CBM and makes a number of contacts with the αC helix of the kinase domain, which we have termed the C-interacting helix. The C-helix and C-interacting helix are oriented approximately anti-parallel with three residues at the N-terminus of the latter (Val-162, Phe-163 and Leu-166) making hydrophobic contacts with Ile-65$_{(C\text{-helix})}$, Ile-61$_{(C\text{-helix})}$ and the aliphatic portion of Lys-62$_{(C\text{-helix})}$. From the middle to the C-terminal end of the C-interacting helix there are a range of polar interactions: Asp-169$_{(C\text{-interacting})}$ with Arg-49$_{(C\text{-helix})}$, and Cys-173$_{(C\text{-interacting})}$ hydrogen bonding with Asp-56$_{(C\text{-helix})}$.

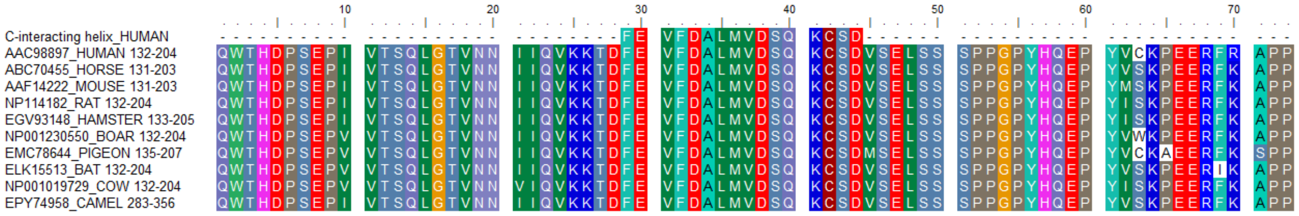

Supplementary Figure 6. Sequence conservation in the C-interacting helix region. Alignment of the AMPK β1 C-interacting helix between different species. We were interested in examining the conservation of this C-interacting helix between various species. To do this, we performed a NCBI Blast search with the C-interacting helix sequence of human AMPK β1 (160-FEVFDALMVDSQKCSD-175) resulting in numerous hits covering over 40 species. The search identified 100% sequence similarity in the β1 isoform between these species. The sequence is also highly conserved between the human AMPK β1 and β2 isoforms (human β2 162-FEVFDALKLDSMESSE-177). A NCBI Blast search using the C-interacting helix sequence of human AMPK β2 resulted in numerous hits covering over 40 species with 100% sequence identity in the β2 isoforms of these species. Taken together, these results suggest that the C-interacting helix is highly conserved across species and suggests an important role of this region in the regulation of AMPK. A few examples are shown in the figure. The sequences were extracted and inputted into the Bioedit program and aligned by ClusterW multiple alignment program.

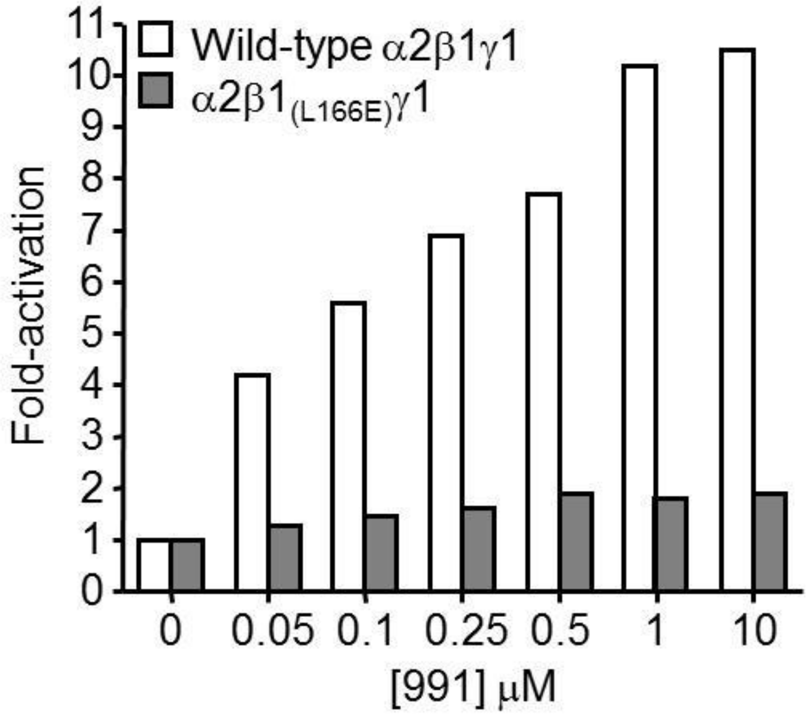

Supplementary Figure 7. Effect of mutating the C-interacting helix on the regulation of AMPK by 991. Mutation of the C-interacting helix and allosteric activation of AMPK. To determine the role of the C-interacting helix in the activation of AMPK by 991, we generated a mutation in a highly conserved Leucine residue (human β1 L166E). This mutant AMPK complex displayed a reduction in the allosteric activation of AMPK by 991. Recombinant AMPK, Wild-type or harbouring the β1 L166E mutation was incubated with varying concentrations of 991 compound (as above) and AMPK activity was determined by the SAMS peptide assay. Results are plotted as the fold activation compared to AMPK incubated without 991 compound and are the data from two independent experiments.

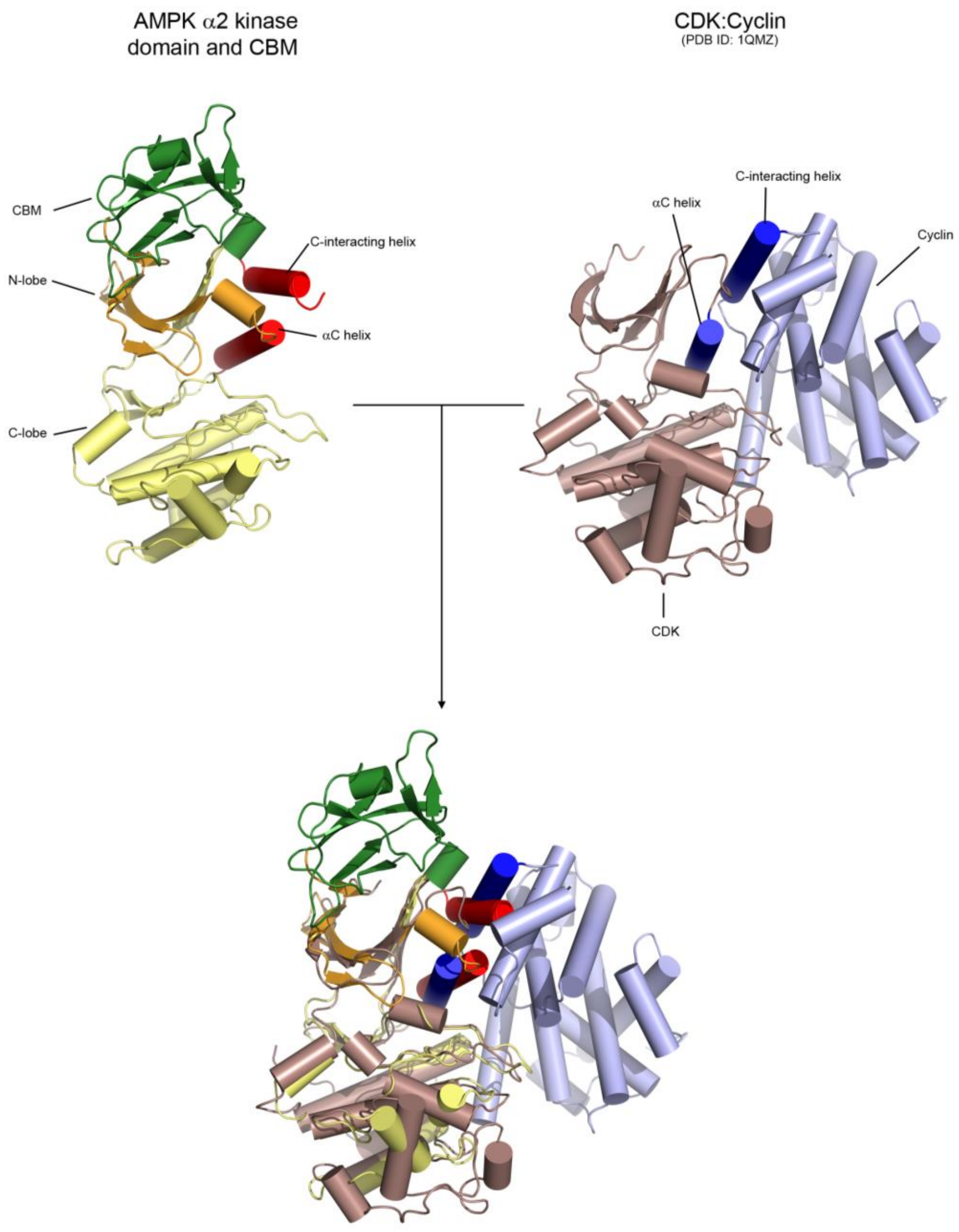

Supplementary Figure 8. Stabilisation of the αC helix during activation of kinases. There are many kinases that are activated by extra domains or separate subunits. One of the ways this is achieved is through stabilisation of the αC helix, thus promoting a conformation that is conducive for efficient ATP binding, phosphate transfer and ADP release. For example, CDKs are activated by cyclin binding as a result of stabilisation of the CDK kinase domain αC helix by helix α5 of the Cyclin protein (PDB ID: 1QMZ, right panel). αC helix (CDK) and C-interacting helix (Cyclin) are coloured blue to highlight these regions. In our new structure of AMPK (left panel), a helix extending from the CBM packs against the α2 kinase domain αC helix (the C-interacting helix and αC helix are coloured red). This interaction is important for mediating the allosteric activation of AMPK by compounds (Supplementary Figure 7). The structures are superposed in the lower panel.

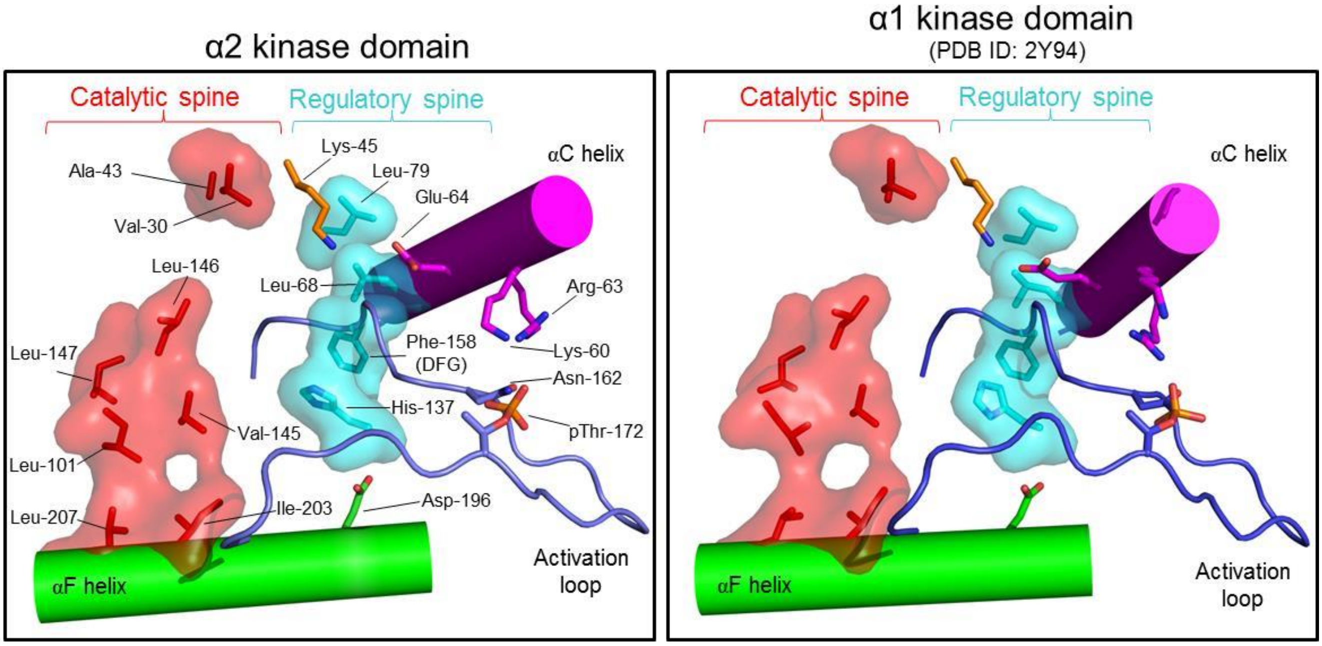

Supplementary Figure 9. Comparison of the catalytic and regulatory spine residues in the AMPK α1 and α2 kinase domains. Comparison of the catalytic and regulatory spine residues in the AMPK α1 and α2 kinase domains. Our activator-bound AMPK structures display many features that are consistent with an active kinase similar to our previous structure (Xiao et al., 2011). The residues that constitute the regulatory and catalytic spines are in the correct positions, including phenylalanine 158 within the DFG motif which adopts the classic 'DFG-in' conformation that is a hallmark of an active kinase (Jura et al., 2011). The catalytic spine (red) and regulatory spine (cyan) residues are shown for the α2 kinase domain (left, our new structure with activator bound) and α1 kinase domain from our previous structure lacking the CBM (right, PDB ID: 2Y94). The αF helix (green) is an essential structural element that allows the assembly of the two hydrophobic spines. The αC helix (magenta) is rotated in the α2 kinase domain to facilitate the DFG flip (see Supplementary Figure 10). The activation loop (blue) is ordered through phosphorylation of Thr-172, and through extensive interactions with the kinase domain (see Supplementary Figure 12) and the regulatory fragment (not shown here). The numbering of the residues is the same in each of the kinase domains (α2, human and α1, rat).

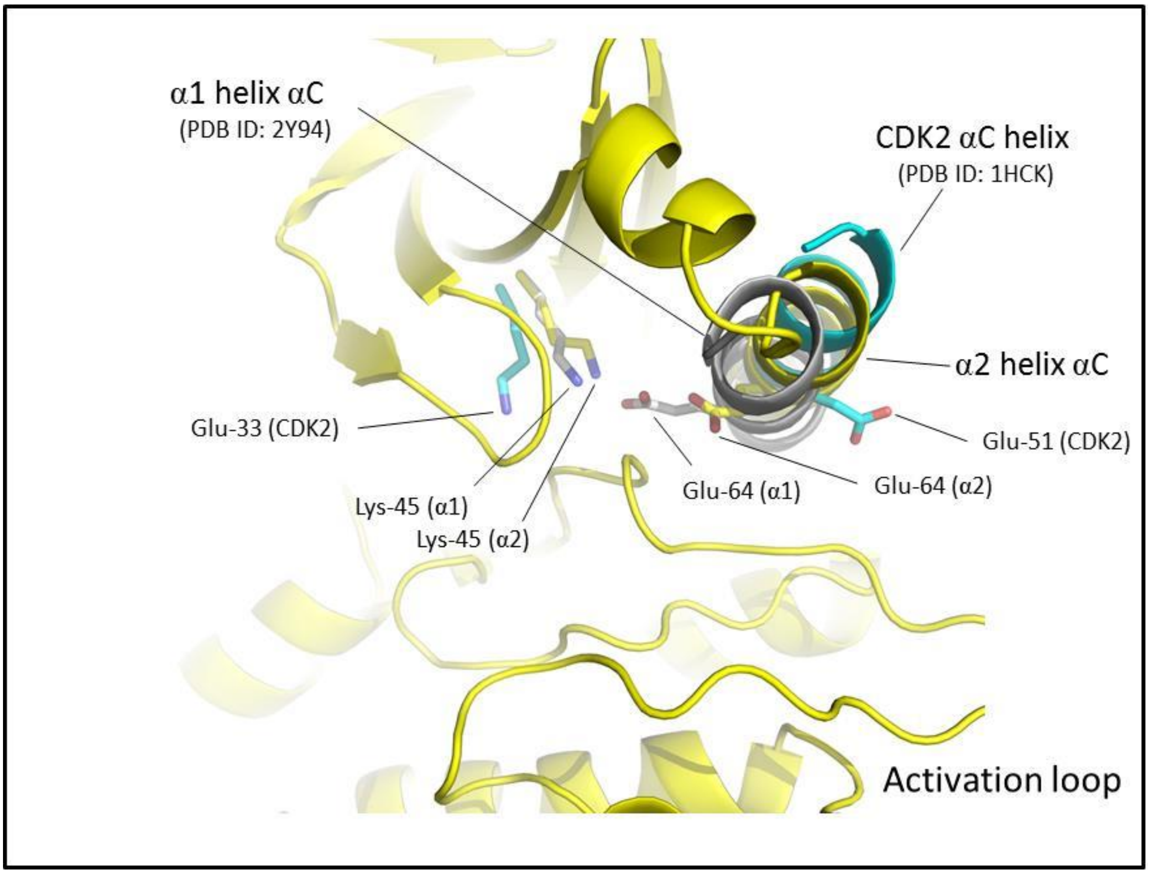

Supplementary Figure 10. Comparison of the αC helix in the kinase domains of AMPK and CDK2. Comparison of the αC helix from AMPK α1 (grey), α2 (yellow) and CDK2 (cyan) kinase domains. The DFG motif is mobile and must flip between a 'DFG-in' (active kinase) and 'DFG-out' (inactive kinase) state so that ATP can bind and ADP can be released (this is the rate limiting step in enzyme catalysis). It has been previously observed that to facilitate the DFG motif flip, the αC helix undergoes a shift such that it leads to breakage of the Lys-Glu ion pair, for example in CDK2 (PDB ID: 1HCK)[50]. The DFG flip is associated with movement of the αC helix as observed in our current α2 kinase domain structure and a CDK2 structure (PDB ID: 1HCK) supporting this hypothesis. Taken together, these multiple observations highlight conformational changes required for an active kinase. The images show the helix αC, the catalytic lysine and catalytic glutamate (coloured according to the αC helix colour; α1 (grey), α2 (yellow) and CDK2 (cyan)).

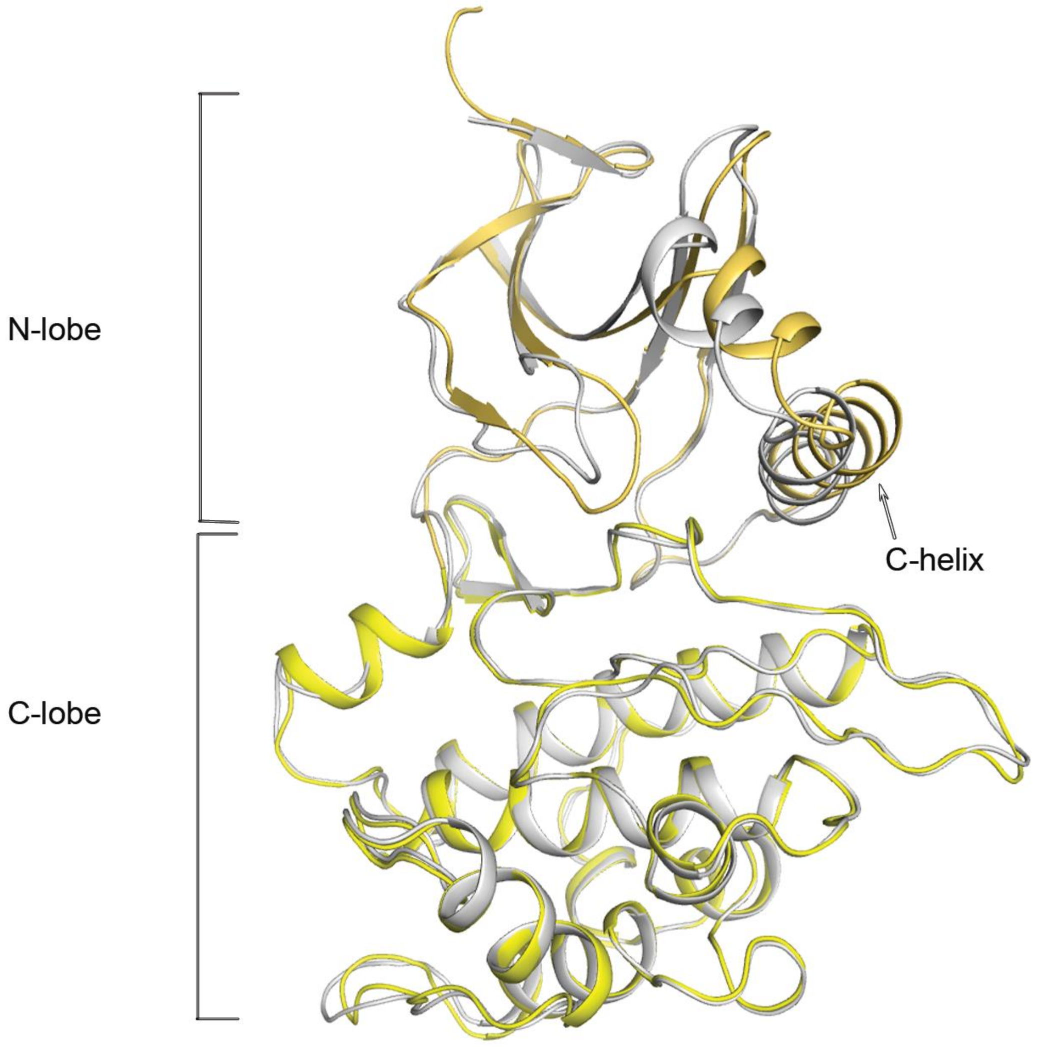

Supplementary Figure 11. Comparison of α1 and α2 kinase structures. Superposition of the kinase domain from AMPK/991 (coloured yellow) and ΔCBM AMPK (coloured grey) based on fitting of the two C-lobes reveals a rigid-body rotation of the N-lobe, with respect to the C-lobe, between the two structures. Also, the C-helix is located further from the activation loop (C-lobe) in the AMPK/991 complex than in ΔCBM AMPK.

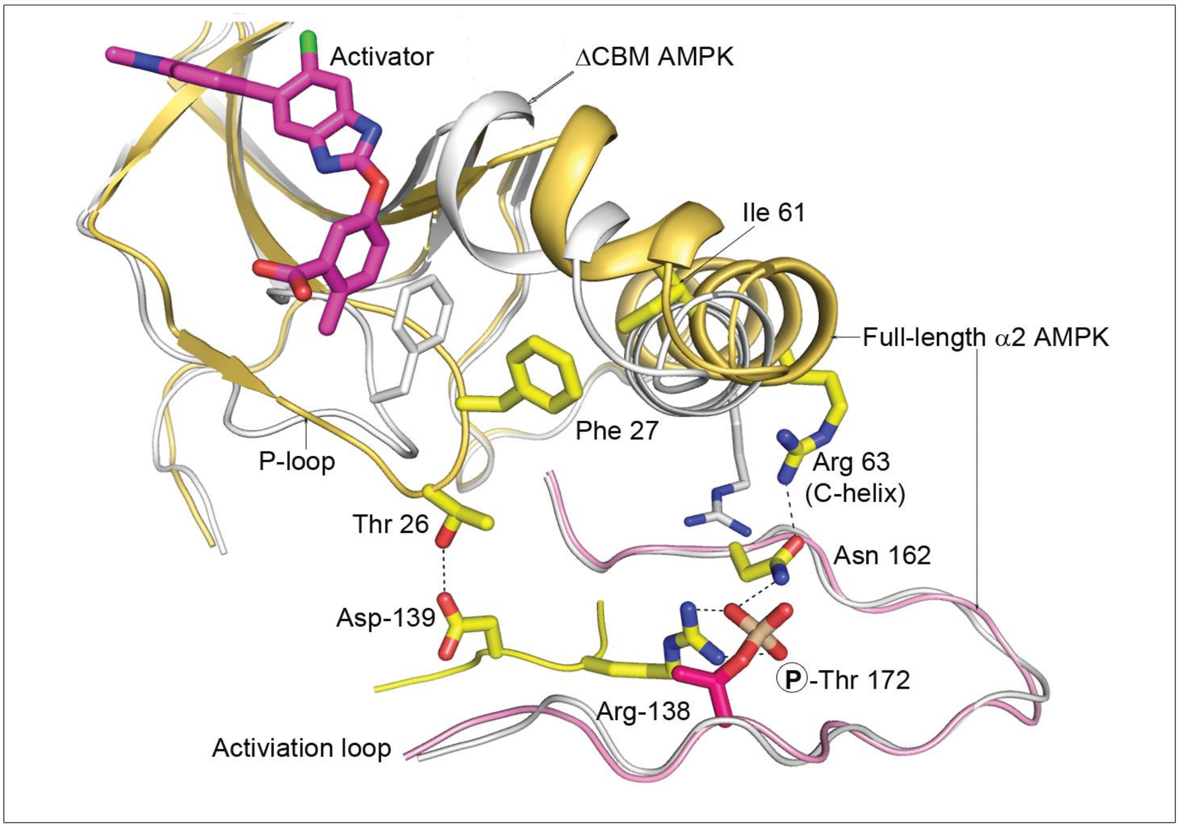

Supplementary Figure 12. Residue interactions in the α1 and α2 kinase structures. Ribbons representation of an overlap of the P- and Activation-loops from the full-length/activator complex (yellow and pink) with those from the ΔCBM AMPK complex (grey). The activator is shown in stick representation with its carbon atoms coloured purple. Only two selected side-chains are shown for the ΔCBM AMPK structure (Phe-27 and Arg-63, with carbon atoms coloured in grey). For the full-length structure additional side-chains are shown that participate in a hydrogen bond network associated the pThr-172 on the activation-loop.

The side chain of Phe-27$_{(P\text{-loop})}$ flips its orientation by about 90 degrees so that it sits in a hydrophobic pocket generated by residues on the C-helix: Leu-47, Ile-52, Leu-55, Val-57 and Ile-61. In concert with the movement of Phe-27$_{(P\text{-loop})}$, Thr-26$_{(P\text{-loop})}$ is positioned more than 3 Å away from its position in the ΔCBM AMPK structure such that it can now form a hydrogen bond with side chain of Asp-139$_{(Kinase\ C\text{-lobe})}$. Perhaps in part due to the interaction with Asp-139$_{(Kinase\ C\text{-lobe})}$, and the movement of the C-helix, a new network of interactions is formed with the phosphate group of pThr-172$_{(Activation\text{-loop})}$. The orientation of the phosphate group enables a bidentate electrostatic interaction with the guanidinium group of Arg-138$_{(Kinase\ C\text{-lobe})}$, as well as a hydrogen bond with Asn-162$_{(Activation\text{-loop})}$ that replaces the previously observed interaction with Arg-63$_{(C\text{-helix})}$ (in ΔCBM). Instead, the side chain of Arg-63$_{(C\text{-helix})}$ hydrogen bonds with the side chain of Asn-162$_{(Activation\text{-loop})}$.

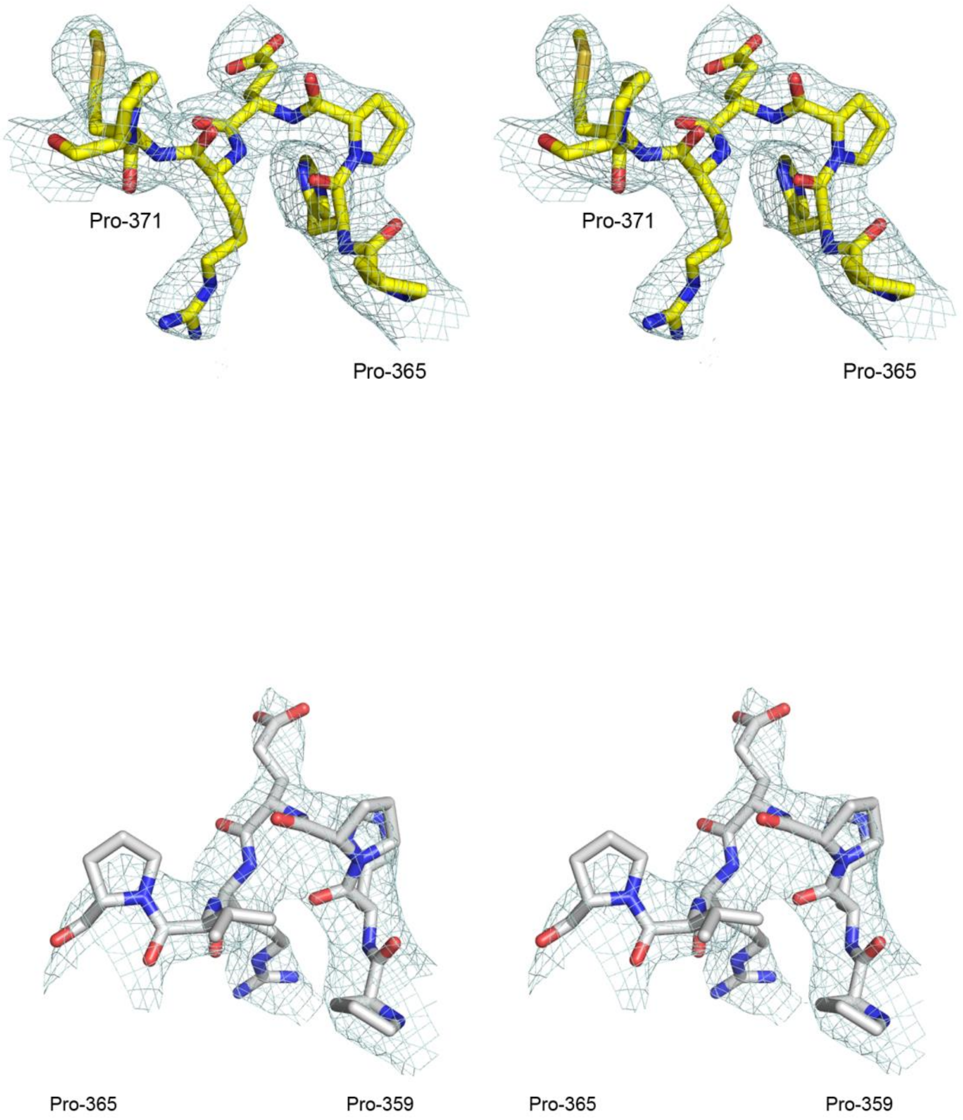

Supplementary Figure 13. Reinterpretation of α1 ΔCBM structure α hook region. The upper panel shows a stereo pair of the portion of electron density map covering the α2 hook region, of the full-length AMPK structured reported here, overlaid on the final model (residues 365-371 Pro-His-Pro-Glu-Arg-Met-Pro). The electron density is from a 2FoFc map contoured at 1 sigma. The lower panel shows the equivalent residues from α1 after rebuilding in the light of the suggestion by Wu and colleagues and the structure of α2 above. The residues are from 359-365 (Pro-His-Pro-Glu-Arg-Val-Pro) and the 2FoFc map is contoured at 1 sigma.

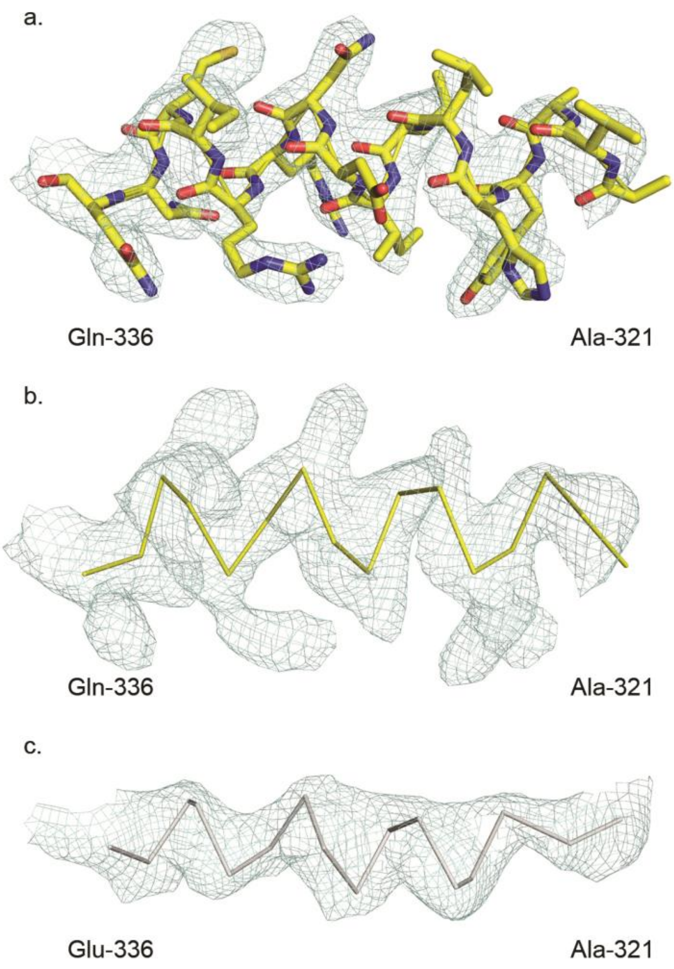

Supplementary Figure 14. Reinterpretation of α1 ΔCBM structure AID region. Chen *et al*. have suggested[51] how their isolated AID crystal structure can be docked onto our earlier ΔCBM structure[28]. This superposition results in reasonably strong, but poorly defined, electron density for the AID α3 helix and poorer density for α1 helix with little interpretable electron density for the other components of the domain. Nonetheless, the relative orientations of the two helices (α1 and α3) are similar between the two structures. In our current structure there is significantly better density for the α3 helix where the sequence register is now convincing, and reasonable main chain, but not side-chain, definition for α1. Again there is not much electron density for other parts of the domain but our new structure supports the interpretation that the α1 and α3 helices of the AID adopt a similar structure between the isolated domain and its conformation in full-length AMPK. The top panel shows helix-3 (residues 321 to 336) from the AID region of the full-length α2 structure together with part of a 2FoFc map contoured at 1 sigma (a). In this map there is sufficient detail to justify the sequence register that has been built. For ease of viewing and comparison with (c), the map from (a) is reproduced in (b) with just the Cα of the helix shown. In the lower panel, the equivalent portion of the electron density map is shown for the α1 ΔCBM structure (c).

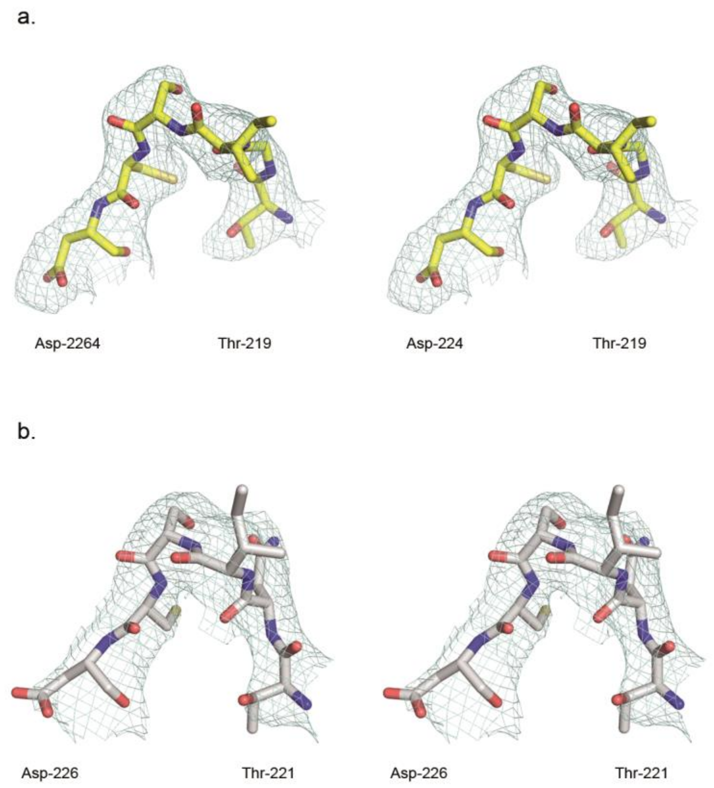

Supplementary Figure 15. Reinterpretation of a loop in the β subunit of the α1 ΔCBM structure. In the new α2 structure the quality of the electron density map is better in many areas and, for example, the sequence register for a loop in the β1 subunit (residues 219-224) is clear (a). In the original α1 ΔCBM structure the quality of the β2 electron density map (b) meant that we could not be sure of this sequence register. We have now been able to reinterpret the build over this loop. Both maps are 2FoFc contoured at 1 sigma.

Using our new structure, and following the suggestion of a different sequence from Wu and colleagues[51], we have reinterpreted these regions of the original α1 ΔCBM structure. The relevant crystallographic statistics are presented in the accompanying table (**Supplementary Table 1**) and new coordinates deposited with the Protein Databank.

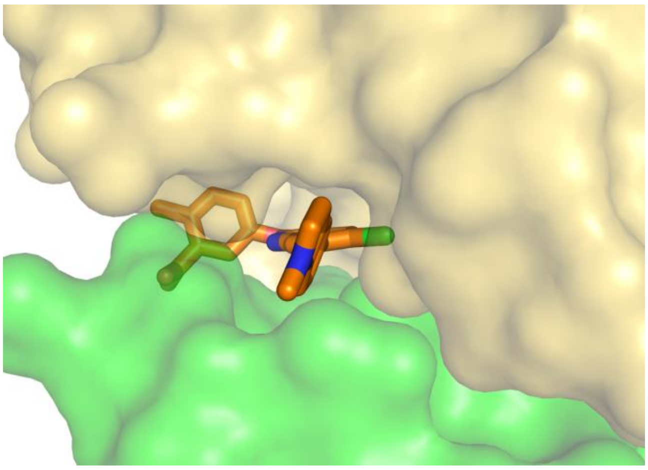

Supplementary Figure 16. CBM/kinase domain interaction. The figure shows space filling representations of the kinase domain (in yellow) and the CBM in green. The 991 compound is shown in stick representation showing that the compound sits in a large cavity between the two domains. In the absence of the activator it would be anticipated that there would be a significantly weaker interaction between the two domains.

Main Manuscript Figure 1C

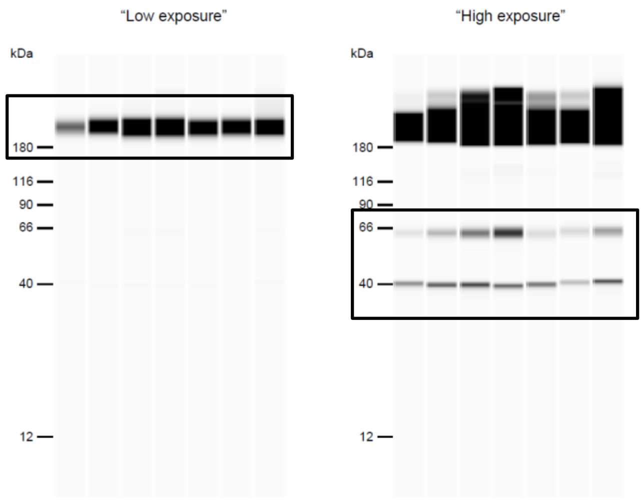

Main Manuscript Figure 1D

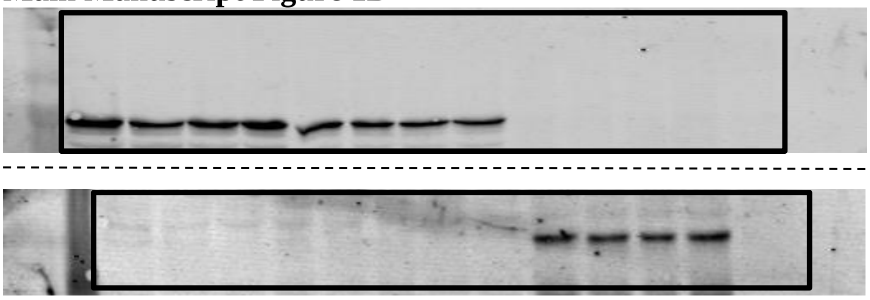

Supplementary Figure S1
pT172 blot

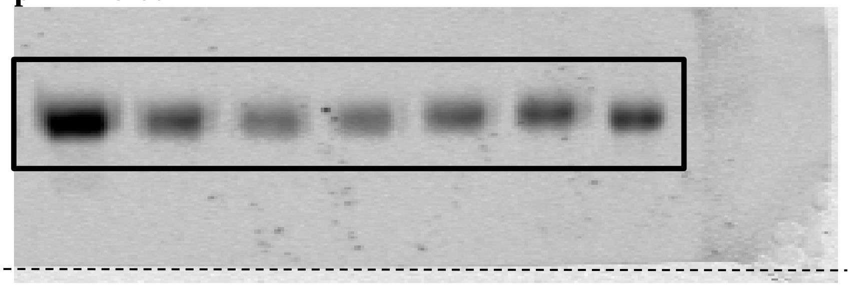

Total α1 blot

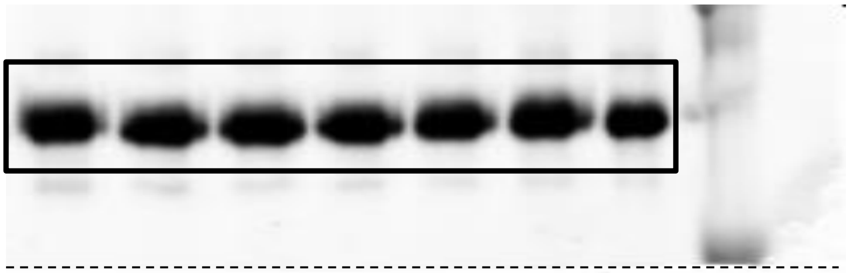

Supplementary Figure 17. Full scan of blots used to generate the figures. Full scan blots used in the manuscript. Boxed regions correspond to the region used to generate the Figures. Dotted lines represent where a blot was cut to allow the use of different antibodies on the same blot.

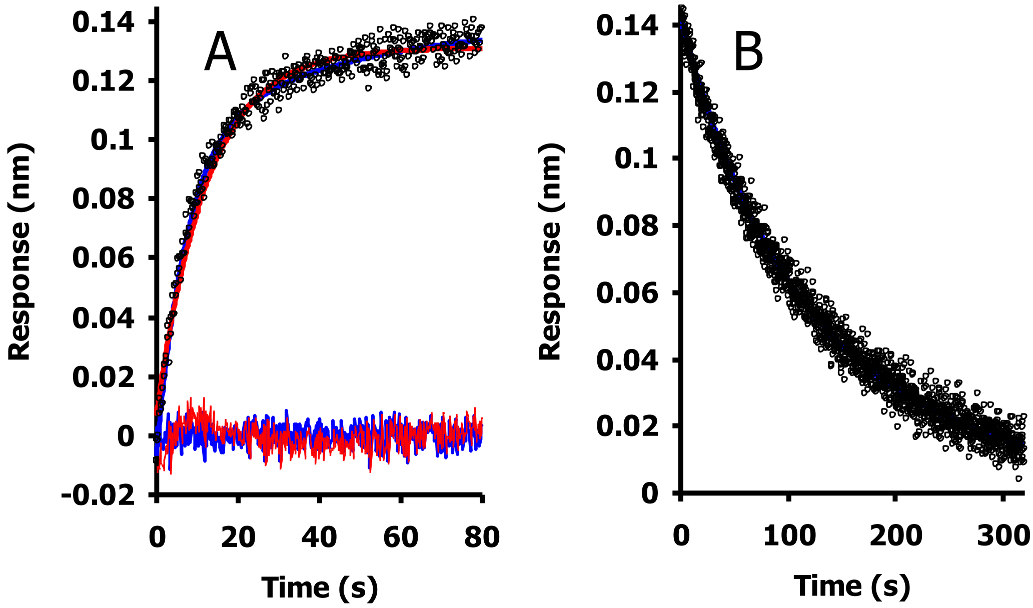

Supplementary Figure 18. Biolayer Interferometry (BLI) measurements and analysis of 991 binding to α1β1γ1. A) Shows the average of 4 traces for the binding of 991 (1.5 μM) to α1β1γ1. B) The figure shows the average of 4 traces for the dissociation of 991 (1.5 μM in the association phase) from α1β1γ1.

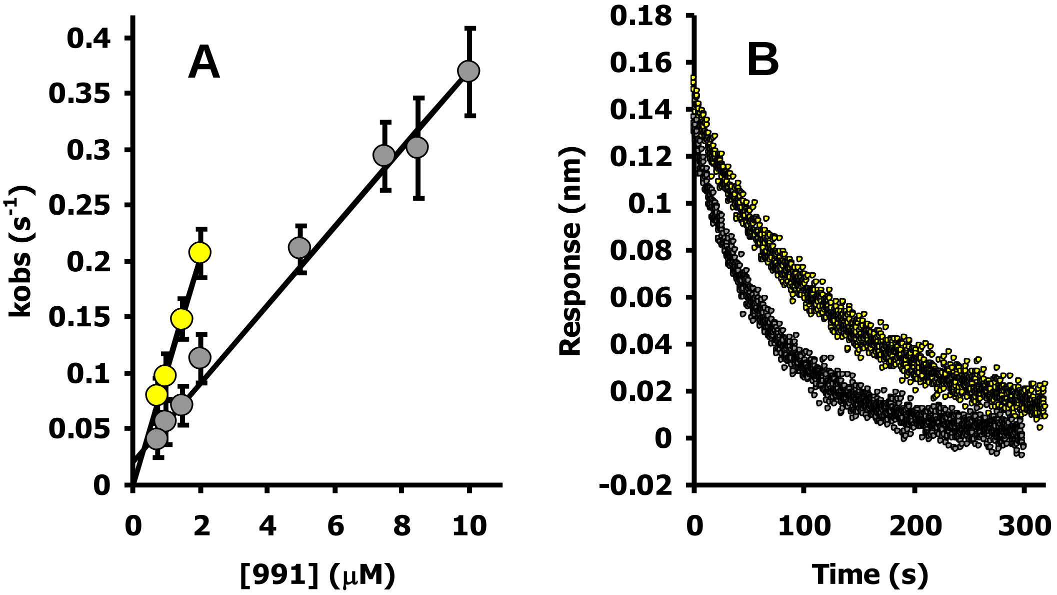

Supplementary Figure 19. Biolayer Interferometry (BLI) measurements and analysis of 991 binding to α1β1γ1 and α1β2γ1. A) The association kinetics for the binding of 991 to α1β1γ1 (shown in yellow) and α1β2γ1 (shown in grey). B) Shows the dissociation of 991 from these AMPK complexes. The analysis is the average of 4 traces.

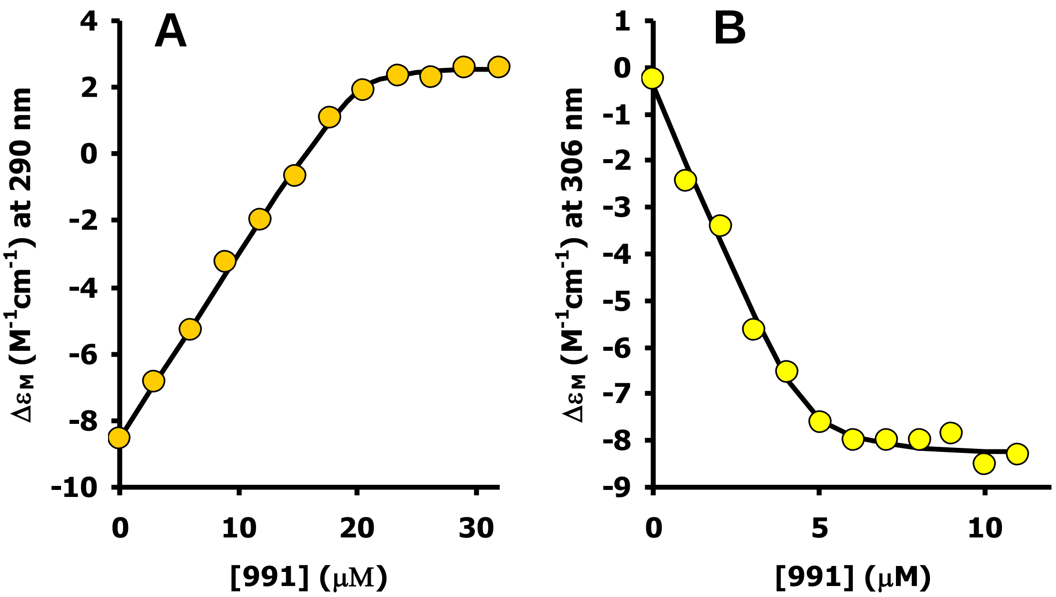

Supplementary Figure 20. Analysis of 991 binding to α1β1γ1 using CD. A) shows the analysis of the signal change at 290 nm after a titration of 20 μM α1β1γ1 with 991 (0 – 32 μM). B) shows the signal changes observed at 306 nm in a titration of 4.65 μM α1β1γ1 with 991 (0 – 11 μM).

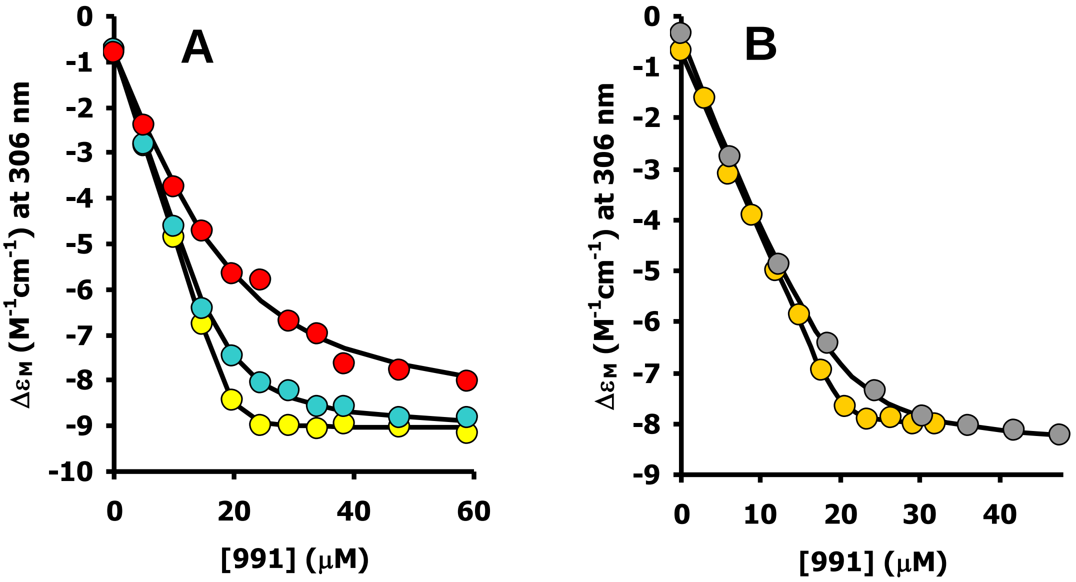

Supplementary Figure 21. Analysis of A-769662 and 991 binding to AMPK using CD. A) shows titrations of α1β1γ1 with 991 in the presence of 0 (yellow), 10 μM (cyan) and 50 μM (red) A-769662. B) compares titrations of α1β1γ1 (yellow) and α1β2γ1 (grey) with 991.

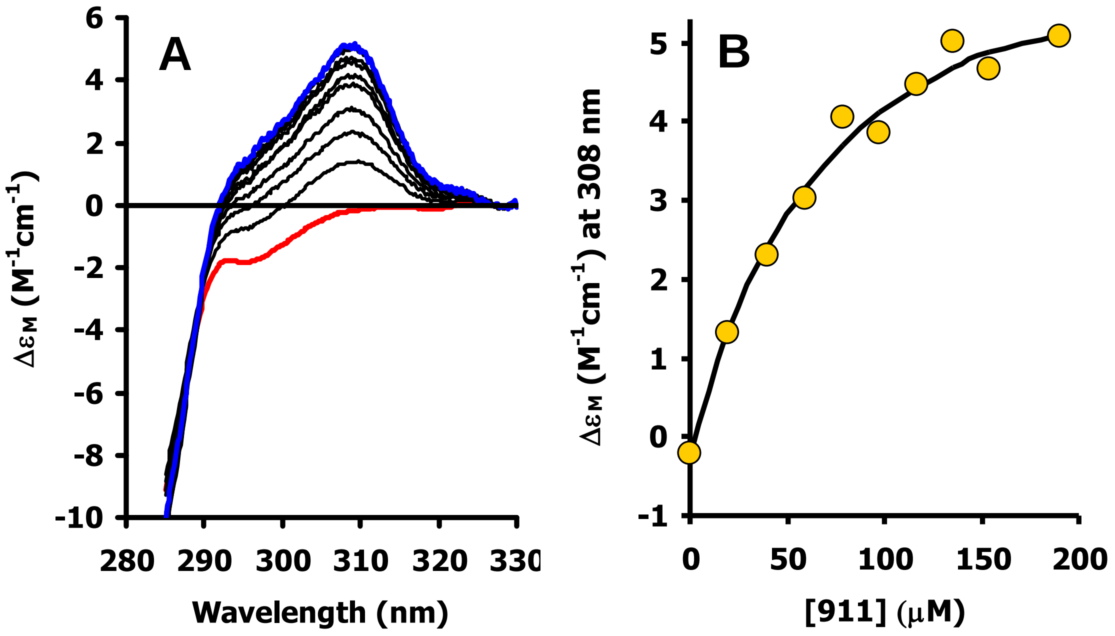

Supplementary Figure 22. Analysis of 991 binding to ΔCBM complex using CD. A) shows a titration of 20 μM ΔCBM complex with 991 (0 – 190 μM) with the protein spectrum in red and the 991:AMPK complex in blue. B) shows the analysis of the signal changes at 308 nm) as a function of 991 concentration (1:1 binding model).

Supplementary Table S1. Data collection and refinement statistics (Molecular replacement)

	ΔCBM AMPK complex
**Data collection**	
Space group	$P4_12_12$
Cell dimensions	
a, b, c (Å)	133.92, 133.92, 141.90
α, β, γ (°)	90, 90, 90
Resolution (Å)	20.0 - 3.24 (3.44 - 3.24)*
R_{sym} or R_{merge}	0.066 (0.506)
$I/\sigma I$	12.1 (2.0)
Completeness (%)	95.8 (93.3)
Redundancy	4.5 (4.5)
**Refinement**	
Resolution (Å)	29.53 – 3.24
No. reflections	19619
R_{work}/ R_{free}	24.2/26.8
No. atoms	
Protein	6348
Ligand/ion	81
Water	
B-factors	
Protein	103.7
Ligand/ion	90.6
Water	
R.m.s deviations	
Bond lengths (Å)	0.008
Bond angles (º)	0.890

One crystal used for the above structure, *Highest resolution shell is shown in parenthesis.

Supplementary Methods.

BLI and CD binding analysis.

Biolayer Interferometry (BLI) measurements:

For a simple 1:1 interaction the time dependence of the biosensor response (R) in the association phase (compound binding) is described by:

$$R = R_{eq}\left(1 - e^{-k_{OBS}t}\right) \qquad [S1]$$

where k_{OBS} is a pseudo first-order rate constant (= k_{on}[C] + k_{off}), [C] is the compound concentration and R_{eq} is the response at equilibrium. Values for k_{on} and k_{off} can then be obtained by plotting k_{OBS} *versus* [C]. In the majority of cases some non-specific binding and/or instrument drift meant that the association phase could not be fit to a single exponential function. For example, Supplementary Figure 18A shows the average of 4 traces for the binding of 991 (1.5 µM) to α1β1γ1.

A single exponential fit (red) gave k_{obs} = 0.09 s^{-1} with non-random residuals and a relatively poor χ^2 value (1.78). A double exponential fit (blue) gave randomly distributed residuals and the χ^2 value improved to 0.96. The fast component of this fit (k_{OBS} = 0.148 s^{-1}) accounted for more than 85% of the total amplitude. The dependence of k_{OBS} on the concentration of 991 for α1β1γ1 (Main text Figure 2A inset) allowed determination of k_{on} (0.103 ± 0.008 $\mu M^{-1}s^{-1}$) from the slope but k_{off} could not be determined. In this case k_{off} was determined from the dissociation phase (when the biosensor is dipped into buffer) because the time dependence of the response is then given by:

$$R = R_0 e^{-k_{off}t} \qquad [S2]$$

where R_0 is the biosensor response prior to the start of the dissociation.

Supplementary Figure 18B shows the average of 4 traces for the dissociation of 991 (1.5 µM in the association phase) from α1β1γ1. A double exponential fit to this curve gave k_{off} = 0.0075 s^{-1} for the component accounting for more than 90% of the total reaction amplitude. A single exponential fit gave a slightly poorer χ^2 value (1.45 compared with 1.04). Averaging all the values for the dissociation phases gave k_{off} = 0.0062 ± 0.0012, corresponding to a K_d of 60 ± 12 nM for the binding of 991 to α1β1γ1.

Analysis of the association kinetics (Supplementary Figure 19A) gave k_{on} = 0.035 ± 0.002 $\mu M^{-1}s^{-1}$ and k_{off} = 0.021 ± 0.03 s^{-1} for α1β2γ1. Analysis of the average of 4 traces for the dissociation of 991 from α1β2γ1 (8.5 µM in the association phase – Supplementary Figure 19B) with a double exponential gave k_{off} = 0.014 s^{-1} for the major component. Averaging all the values for the dissociation phases gave k_{off} = 0.018 ± 0.003. This gives a K_d of 514 ± 90 nM for the binding of 991 to α1β2γ1. The lower affinity for this construct therefore derives from a decreased association rate constant and an increased dissociation rate constant.

CD Measurements:

Figure 2 (main text) shows a titration of 20 µM α1β1γ1 with 991 (0 – 32 µM). The spectrum of the 991:AMPK complex (shown in blue) is very different from the spectrum of the protein (shown in red). Since uncomplexed 991 has no detectable CD signal at the concentrations employed here the change observed upon complex formation must result from changes in the spectrum of the protein and/or the 991. There could be a 991-induced change in the tertiary structure of the protein that affects one or more tryptophans. However, since binding of 991 does not affect the tryptophan emission spectrum of the protein, a more likely explanation is that the 991 immobilized in the asymmetric environment of the protein develops a strong CD signal.

The precise origin of the CD signal change is unimportant in binding studies and the titrations of the AMPK constructs (P) with drug (D: 991) were therefore fitted to the following equation:

$$\Delta\varepsilon_{OBS} = \Delta\varepsilon_{P}[P] + \Delta\varepsilon_{PD}[PD] \quad [S3]$$

where $\Delta\varepsilon_{OBS}$, $\Delta\varepsilon_{P}$, and $\Delta\varepsilon_{PD}$ are the observed CD extinction coefficient and the CD extinction coefficients for P and PD. A value for the dissociation constant for drug binding ($K_{d,D}$) was obtained from a nonlinear least squares fit to this equation with concentrations calculated by solving equation (S4):

$$[PD]^2 - [PD](K_{d,D} + P_T + D_T) + K_T D_T = 0 \quad [S4]$$

where the subscript T denotes total concentrations.

Analyzing the signal changes at 306 nm (Figure 2B main text inset) as a function of 991 concentration (1:1 binding model) gave a K_d of 78 ± 26 nM. As expected, analysis of the signal change at 290 nm (Supplementary Figure 20A) gave a consistent K_d (105 ± 37 nM). Because these are 'close to stoichiometric' titrations we also performed a series of further measurements using lower overall concentrations. Supplementary Figure 20B shows the signal changes observed at 306 nm in a titration of 4.65 µM α1β1γ1 with 991 (0 – 11 µM) and analysis again yielded a consistent K_d (72 ± 32 nM).

Although A-769662 binding to the constructs used here does not induce any change in the aromatic CD region it was possible to determine K_d values by studying the binding of 991 to AMPK constructs in the presence of a fixed concentration of A-769662. Supplementary Figure 21A shows titrations of α1β1γ1

with 991 in the presence of 0 (yellow), 10 µM (cyan) and 50 µM (red) A-769662. These curves were also fitted to equation (S3) but with concentrations calculated by solving the following equation with $K_{d,D}$ fixed at the value determined from the direct titration:

$$[P]^3 + [P]^2(-P_T + K_{d,D} + K_{d,A} + D_T + A_T) + [P](-P_T K_{d,D} - P_T K_{d,A} + K_{d,D}K_{d,A} + D_T K_{d,A} + A_T K_{d,D}) - P_T K_{d,D} K_{d,A} = 0 \quad \text{[S5]}$$

where A_T and $K_{d,A}$ are the total A-769662 concentration and the K_d respectively.

The analyses gave K_ds for A-769662 of 0.62 ± 0.18 µM (10 µM) and 0.85 ± 0.23 µM (50 µM), which are in reasonable agreement with the values determined using BLI (0.51 ± 0.14 µM for A-769662 binding to α1β1γ1).

Constructs containing β2 bind more weakly than those containing β1 as shown in Supplementary Figure 21B which compares titrations of α1β1γ1 (yellow) and α1β2γ1 (grey) with 991. The K_d value for α1β2γ1 determined from this titration was 1.18 ± 0.31 µM. Constructs containing α2 bound 991 with similar affinities to those determined for the corresponding α1 constructs (Table 2).

The ΔCBM (α1β1(185-270)γ1) complex binds 991 much more weakly, and the spectrum of the complex formed is very different from the corresponding complexes formed with the full length proteins. Supplementary Figure 22A shows a titration of 20 µM ΔCBM complex with 991 (0 – 190 µM) with the protein spectrum in red and the 991:AMPK complex in blue. Analyzing the signal changes at 308 nm (Supplementary Figure 22B) as a function of 991 concentration (1:1 binding model) gave a K_d of 51 ± 11 µM.

Supplementary Reference

59. Bookser, B.C. *et al.* Novel cyclic benzimidazole derivatives useful anti-diabetic agents. WO2010/036613 filed 21 Sep. 2009, and issued 1 Apr. 2010.
